# Obesity and Its Metabolic Complications: The Role of Adipokines and the Relationship between Obesity, Inflammation, Insulin Resistance, Dyslipidemia and Nonalcoholic Fatty Liver Disease

**DOI:** 10.3390/ijms15046184

**Published:** 2014-04-11

**Authors:** Un Ju Jung, Myung-Sook Choi

**Affiliations:** Center for Food and Nutritional Genomics Research, Kyungpook National University, 1370 Sankyuk Dong Puk-ku, Daegu 702-701, Korea; E-Mail: jungunju@naver.com

**Keywords:** obesity, inflammation, insulin resistance, dyslipidemia, nonalcoholic fatty liver disease, adipose tissue, adipokine

## Abstract

Accumulating evidence indicates that obesity is closely associated with an increased risk of metabolic diseases such as insulin resistance, type 2 diabetes, dyslipidemia and nonalcoholic fatty liver disease. Obesity results from an imbalance between food intake and energy expenditure, which leads to an excessive accumulation of adipose tissue. Adipose tissue is now recognized not only as a main site of storage of excess energy derived from food intake but also as an endocrine organ. The expansion of adipose tissue produces a number of bioactive substances, known as adipocytokines or adipokines, which trigger chronic low-grade inflammation and interact with a range of processes in many different organs. Although the precise mechanisms are still unclear, dysregulated production or secretion of these adipokines caused by excess adipose tissue and adipose tissue dysfunction can contribute to the development of obesity-related metabolic diseases. In this review, we focus on the role of several adipokines associated with obesity and the potential impact on obesity-related metabolic diseases. Multiple lines evidence provides valuable insights into the roles of adipokines in the development of obesity and its metabolic complications. Further research is still required to fully understand the mechanisms underlying the metabolic actions of a few newly identified adipokines.

## 1. Introduction

**Figure 1.**
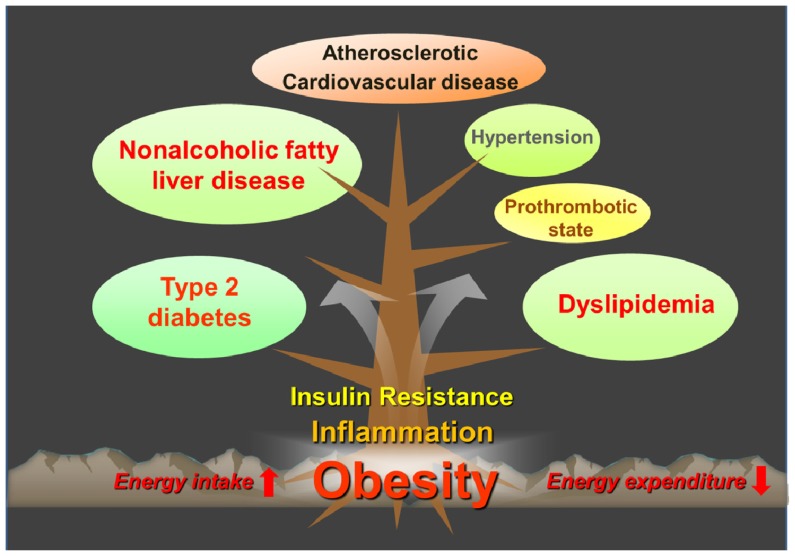
Concept of metabolic syndrome.

The worldwide prevalence of obesity and its metabolic complications have increased substantially in recent decades. According to the World Health Organization, the global prevalence of obesity has nearly doubled between 1980 and 2008, and more than 10% of the adults aged 20 and over is obese in 2008 [1]. Projections based on the current obesity trends estimate that there will be 65 million more obese adults in the USA and 11 million more obese adults in the UK by 2030, consequently accruing an additional 6–8.5 million cases of diabetes, 5.7–7.3 million cases of heart disease and stroke for USA and UK combined [2]. The increased prevalence in obesity is also associated with increasing prevalence of nonalcoholic fatty liver disease (NAFLD). Among the Americas, the prevalence of NAFLD is highest in the USA, Belize and Barbados and Mexico, which have a high prevalence of obesity [3]. Obesity, especially abdominal obesity, is one of the predominant underlying risk factors for metabolic syndrome [4]. Obesity increases the risk of developing a variety of pathological conditions, including insulin resistance, type 2 diabetes, dyslipidemia, hypertension and NAFLD (Figure 1). Accumulating evidence suggests that chronic inflammation in adipose tissue may play a critical role in the development of obesity-related metabolic dysfunction [5–7].

Adipose tissue has been recognized as an active endocrine organ and a main energy store of the body [8]. Excess adiposity and adipocyte dysfunction result in dysregulation of a wide range of adipose tissue-derived secretory factors, referred to as adipokines, which may contribute to the development of various metabolic diseases via altered glucose and lipid homeostasis as well as inflammatory responses [9,10]. In addition, excess fat accumulation promotes the release of free fatty acids into the circulation from adipocytes, which may be a critical factor in modulating insulin sensitivity [11,12]. However, plasma free fatty acid levels do not increase in proportion to the amount of body fat, since their basal adipose tissue lipolysis per kilogram of fat is lower in obese subjects than in lean subjects [13]. This finding has been supported by other studies of adipocytes from obese subjects [14,15] and it was associated with down-regulation of hormone sensitive lipase and adipose triglyceride lipase, key enzymes involved in intracellular degradation of triglycerides [14,16–18]. Thus, Karpe *et al.* [19] have recently suggested that the link between circulating free fatty acid levels and insulin sensitivity *in vivo* is needed to further elucidate this complicated relationship.

In this review, we will first discuss the critical role of adipose tissue for health and as a repository of free fatty acids. We will also review how the dysregulation of free fatty acids and inflammatory factors released by enlarged adipose tissue is associated with the pathogenesis of metabolic syndrome (insulin resistance, dyslipidemia and NAFLD). In particular, we will focus on the imbalance of pro-inflammatory and anti-inflammatory molecules secreted by adipose tissue which contribute to metabolic dysfunction.

## 2. Function of Adipose Tissue

Adipose tissue is the major site for storage of excess energy in the form of triglycerides, and it contains multiple cell types, including mostly adipocytes, preadipocytes, endothelial cells and immune cells. During positive energy balance, adipose tissue stores excess energy as triglycerides in the lipid droplets of adipocytes through an increase in the number of adipocyte (hyperplasia) or an enlargement in the size of adipocytes (hypertrophy) [20]. The number of adipocytes is mainly determined in childhood and adolescence and remains constant during adulthood in both lean and obese subjects, even after marked weight loss [21]. Hence, an increase in fat mass in adulthood can primarily be attributed to hypertrophy. However, recent study has reported that normal-weight adults can expand lower-body subcutaneous fat, but not upper-body subcutaneous fat, via hyperplasia in response to overfeeding [22], suggesting hyperplasia of adipocytes can also occur in adulthood. Although overall obesity is associated with metabolic diseases, adipose tissue dysfunction caused by hypertrophy has been suggested to play an important role in the development of metabolic diseases such as insulin resistance [23–25]. In contrast to positive energy balance states, when energy is needed between meals or during physical exercise, triglycerides stored in adipocytes can be mobilized through lipolysis to release free fatty acids into circulation and the resulting free fatty acids are transported to other tissues to be used as an energy source. It is generally accepted that free fatty acids, a product of lipolysis, play a critical role in the development of obesity-related metabolic disturbances, especially insulin resistance. In obesity, free fatty acids can directly enter the liver via the portal circulation, and increased levels of hepatic free fatty acids induce increased lipid synthesis and gluconeogenesis as well as insulin resistance in the liver [26]. High levels of circulating free fatty acids can also cause peripheral insulin resistance in both animals and humans [26,27]. Moreover, free fatty acids serve as ligands for the toll-like receptor 4 (TLR4) complex [28] and stimulate cytokine production of macrophages [29], thereby modulating inflammation of adipose tissue which contributes to obesity-associated metabolic complications. However, circulating free fatty acid concentrations do not increase in proportion to fat mass and do not predict the development of metabolic syndrome [30–33], although many studies suggest a relationship between the release of free fatty acids from adipose tissue and obesity-related metabolic disorders.

**Figure 2.**
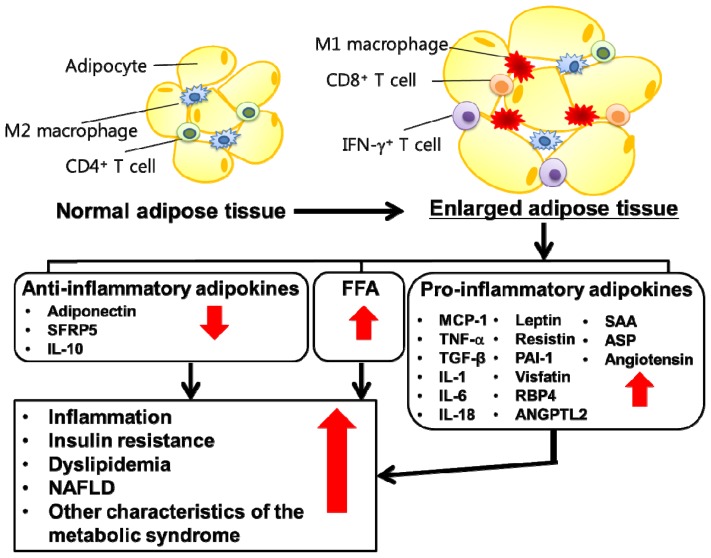
Secretion of inflammatory adipokines from adipose tissue in obese state. In obese state, the enlarged adipose tissue leads to dysregulated secretion of adipokines and increased release of free fatty acids. The free fatty acids and pro-inflammatory adipokines get to metabolic tissues, including skeletal muscle and liver, and modify inflammatory responses as well as glucose and lipid metabolism, thereby contributing to metabolic syndrome. In addition, obesity induces a phenotypic switch in adipose tissue from anti-inflammatory (M2) to pro-inflammatory (M1) macrophages. On the other hand, the adipose production of insulin-sensitizing adipokines with anti-inflammatory properties, such as adiponectin, is decreased in obese state. The red arrows indicate increased (when pointing upward) or decreased (when pointing downward) responses to obesity. ANGPTL, angiopoietin-like protein; ASP, acylation-stimulating protein; IL, interleukin; MCP-1, monocyte chemotactic protein; NAFLD, nonalcoholic fatty liver disease; PAI-1, plasminogen activator inhibitor-1; RBP4, retinol binding protein 4; SAA, serum amyloid A; SFRP5, secreted frizzled-related protein 5; TGF-β, Transforming growth factor-β; TNF-α, tumor necrosis factor-α.

Adipose tissue also has a major endocrine function secreting multiple adipokines (including chemokines, cytokines and hormones) (Figure 2). Many of the adipokines are involved in energy homeostasis and inflammation, including chemokines and cytokines. In the obese state, the adipocyte is integral to the development of obesity-induced inflammation by increasing secretion of various pro-inflammatory chemokines and cytokines [34,35]. Many of them, including monocyte chemotactic protein (MCP)-1, tumor necrosis factor (TNF)-α, interlukin (IL)-1, IL-6 and IL-8, have been reported to promote insulin resistance [36–39]. Moreover, the macrophage content of adipose tissue is positively correlated with both adipocyte size and body mass, and expression of pro-inflammatory cytokines, such as TNF-α, is mostly derived from macrophages rather than adipocytes [40]. Along with the increased number of macrophages in adipose tissue, obesity induces a phenotypic switch in these cells from an anti-inflammatory M2 polarization state to a pro-inflammatory M1 polarization state [41]. The accumulation of M1 macrophages in adipose tissue has been shown to result in secretion of a variety of pro-inflammatory cytokines and chemokines that potentially contribute to obesity-related insulin resistance [5,42]. In contrast, M2-polarized macrophages participate in remodeling of adipose tissue, including clearance of dead or dying adipocytes and recruitment and differentiation of adipocyte progenitors [43]. Decreased adipose macrophage infiltration or macrophage ablation reduces expression of inflammatory cytokines in adipose tissue and improves insulin sensitivity in diet-induced obese mice [44–47]. Furthermore, weight loss decreases macrophage infiltration and pro-inflammatory gene expression in adipose tissue in obese subjects [48,49]. In addition to M1 macrophages, levels of multiple pro-inflammatory immune cells, such as interferon (IFN)-γ^+^ T helper type 1 cells and CD8^+^ T cells, are increased in adipose tissue in obesity [50]. In contrast, secretion of insulin-sensitizing adiponectin is reduced in obese subjects [51].

## 3. Obesity and Insulin Resistance

**Figure 3.**
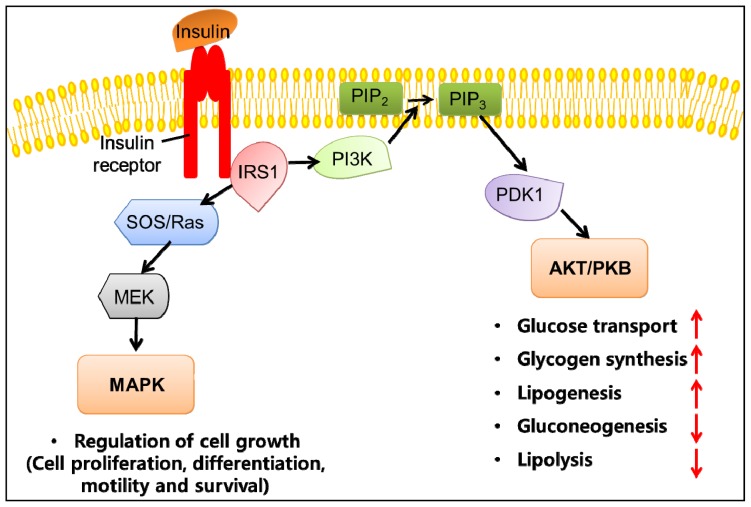
Schematic view of insulin signaling pathway in adipose tissue. Binding of insulin to its receptor on adipocytes triggers the phosphorylation and activation of insulin receptor substrate, which forms a docking site for phosphatidylinositol 3-kinase (PI3K) at the membrane. When docked, PI3K converts phosphatidylinositol 4,5-bisphosphate to phosphatidylinositol 3,4,5-trisphosphate, a second messenger that activates phosphoinositide-dependent protein kinase 1 and recruits Akt (also known as protein kinase B, PKB) to the cell membrane. Consequently, PI3K-AKT/PKB signaling pathway regulates metabolic processes. The red arrows indicate up-regulation (when pointing upward) or down-regulation (when pointing downward) in response to PI3K-AKT/PKB signaling pathway. The Ras-mitogen-activated protein kinase pathway leads to the activation of genes which are involved in cell growth, thereby promoting inflammation and atherogenesis. IRS-1, insulin receptor substrate; MAPK, mitogen-activated protein kinase; PDK, phosphoinositide-dependent protein kinase 1; PI3K, phosphatidylinositol 3-kinase; PIP2, phosphatidylinositol 4,5-bisphosphate; PIP3, phosphatidylinositol 3,4,5-trisphosphate; PKB, protein kinase B.

Insulin resistance is an integral feature of metabolic syndrome and is a major predictor of the development of type 2 diabetes [52]. It has long been recognized that obesity is associated with type 2 diabetes, and the major basis for this link is the ability of obesity to induce insulin resistance. Insulin resistance is defined as the decreased ability of tissues to respond to insulin action. Adipose tissue is one of the insulin-responsive tissues, and insulin stimulates storage of triglycerides in adipose tissue by multiple mechanisms, including promoting the differentiation of preadipocytes to adipocytes, increasing the uptake of glucose and fatty acids derived from circulating lipoproteins and lipogenesis in mature adipocytes, and inhibiting lipolysis [53]. The metabolic effects of insulin are mediated by a complex insulin-signaling network (Figure 3). Insulin signaling is initiated when insulin binds to its receptor on the cell membrane, leading to phosphorylation/activation of insulin receptor substrate (IRS) proteins that are associated with the activation of two main signaling pathways: the phosphatidylinositol 3-kinase (PI3K)-AKT/protein kinase B (PKB) pathway and the Ras-mitogen-activated protein kinase (MAPK) pathway. The PI3K-AKT/PKB pathway is important for most metabolic actions of insulin. IRS-1, which is phosphorylated by the insulin receptor, activates PI3K by binding to its SH2 domain. PI3K generates phosphatidylinositol-(3,4,5)-triphosphate, a lipid second messenger, which activates several phosphatidylinositol-(3,4,5)-triphosphate-dependent serine/threonine kinases, including AKT/PKB. Ultimately, these signalling events result in the translocation of glucose transporter 4 to the plasma membrane, leading to an increase in adipocyte glucose uptake. The MAPK pathways are not implicated in mediating metabolic actions of insulin but rather in stimulating mitogenic and growth effects of insulin. In the adipose tissue, insulin also has an anti-lipolytic effect, whereby the activation of PI3K stimulates phosphodiesterase-3 so that more adenosine 3′,5′-cyclic monophosphate is hydrolyzed in adipocytes, which in turn limits the release of fatty acids from adipocytes. In addition, the transcription factors, including adipocyte determination and differentiation factor 1/sterol regulatory element-binding protein-1c (SREBP1-c), regulate the expression of multiple genes that are responsible for adipocyte differentiation, lipogenesis and fatty acid oxidation.

Evidence has suggested a role for adipose tissue in the development of insulin resistance. As discussed in the preceding text, free fatty acids and various adipokines released from adipose tissue have been involved in abnormal insulin signaling. It has been suggested that fatty acids and their metabolites, such as acyl-coenzyme A, ceramides and diacyglycerol, can impair insulin signaling by promoting protein kinases such as protein kinase C, MAPK, c-Jun *N*-terminal kinase (JNK), and the inhibitor of nuclear factor κB kinase β [54]. Saturated fatty acids, but not unsaturated fatty acids, induce the synthesis of ceramide, and inhibition of ceramide synthesis ameliorates saturated fatty acids-induced insulin resistance [55]. TNF-α also promotes ceramide accrual by activating sphingomyelinase, an enzyme that catalyzes the hydrolysis of sphingomyelin to ceramide [56], and ceramide mediates TNF-α-induced insulin resistance in adipocytes [57]. Haus *et al.* [58] reported that plasma ceramide levels are elevated in obese subjects with type 2 diabetes and it contributes to insulin resistance by activating inflammatory mediators, such as TNF-α. Thus, ceramide has been regarded as mediator linking several metabolic stresses (*i.e.*, TNF-α and saturated fatty acids, but not unsaturated fatty acids) to the induction of insulin resistance [55,57], although the role of TNF-α in insulin resistance is somewhat controversial [59]. Obese subjects had greater whole body free fatty acids rates of appearance in plasma compared with lean subjects [60], and a sustained reduction in plasma free fatty acids levels after treatment of lipolysis inhibitor was associated with an improvement of insulin sensitivity in diabetic obese subjects [61]. The anti-lipolysis drug also decreased fasting plasma free fatty acids levels in lean control, obese nondiabetic, obese subjects with impaired glucose tolerance, and the lowering of plasma free fatty acids levels improved insulin resistance and glucose tolerance in obese subjects, regardless of the degree of their preexisting insulin resistance [62]. Recently, Girousse *et al.* [63] reported that a decrease in adipose tissue lipolysis improved insulin tolerance and glucose metabolism without altering fat mass. Obesity-induced increases in lipolysis not only increases local extracellular lipid concentrations but also derives accumulation of macrophages in adipose tissue [64], which is associated with systemic hyperinsulinemia and insulin resistance in obese subjects [65]. In fact, macrophage recruitment was increased with fat mass [54], and the phenotype of adipose macrophages and recruitment of macrophages and other immune cells to the adipose tissue play important roles in the development of obesity-related insulin resistance [66].

Obesity-induced insulin resistance is also associated with increased secretion of cytokines and other bioactive substances from adipose tissue as well as the number of adipose macrophages. In the adipose tissue of obese humans and animals, there are a large number of macrophages infiltrations, and this recruitment is linked to the pathogenesis of obesity-induced inflammation and insulin resistance [5,40]. The production of most inflammatory factors by adipose tissue is also increased in the obese state and promotes obesity-linked metabolic diseases [67,68]. Adipocytes and immune cells (primarily macrophages) in the adipose tissue are the primary sources of many inflammatory proteins [67,68]. There are two types of inflammatory proteins: pro-inflammatory and anti-inflammatory. A number of pro-inflammatory proteins, including MCP-1, TNF-α, IL-6, IL-18, leptin, resistin, plasminogen activator inhibitor (PAI)-1, visfatin, retinol binding protein 4 (RBP4) and angiopoietin-like protein 2 (ANGPTL2), are described in more detail in the following text. Additionally, we briefly discuss the metabolic properties of two anti-inflammatory adipokines, adiponectin and secreted frizzled-related protein 5 (SFRP5). There are discrepancies between preclinical studies and clinical trials regarding some adipokines, including TNF-α, resistin and SFRP5. Although the cause of discrepancy between preclinical studies and clinical trials is unclear, it may be due to a number of factors including the discrepancies on the species (e.g., difference in the tissue compositions and gene profiles between animals and humans), outcome measures, pre-morbid conditions and treatment methods. In addition, considering the wide spectrum of pro- and anti-inflammatory adipokines, which are altered in obesity, it is likely that crosstalk of many adipokines rather than a single adipokine in adipose tissue and other tissues may be involved in the metabolic dysregulation. Further studies are still required to clarify their roles in various conditions.

## 4. Role of Adipose Tissue-Produced Adipokines in Insulin Resistance

### 4.1. CCL2/MCP-1 and Other Chemokines

Chemokines play a major role in selectively recruiting monocytes, neutrophils, and lymphocytes and in inducing chemotaxis, and chemokines and their receptors are highly expressed in human visceral and subcutaneous adipose tissue in obesity [69]. C-C motif chemokine ligand 2/macrophage chemoattractant protein-1 (CCL2/MCP-1) is one of the key chemokines that regulate migration and infiltration of monocytes/macrophages. It initiates adipose inflammation by attracting inflammatory cells from the blood stream into adipose tissue [66,70]. CCL2/MCP-1 is expressed by adipocytes and circulating levels of CCL2/MCP-1 correlate with adiposity. Over-expression of *CCL2/MCP-1* in adipose tissue increases macrophage recruitment and worsens the metabolic phenotype [71,72], whereas a deficiency of *CCL2/MCP-1* or its receptor *CCR2* reduces pro-inflammatory macrophages accumulation in adipose tissue and provides protection from insulin resistance as well as hepatic steatosis [45,71,73]. Recently, Meijer *et al*. [74] reported that adipocyte-derived CCL2/MCP-1 can stimulate inflammation independently of macrophages/leukocytes in human adipose tissue, although many cells in adipose tissue, including adipocyte and macrophages/leukocytes, produce CCL2/MCP-1. In a large cohort of Caucasians, circulating CCL2/MCP-1 was increased in type 2 diabetes subjects and presence of the *MCP-1* G-2518 allele was associated with decreased plasma CCL2/MCP-1 levels as well as prevalence of insulin resistance and type 2 diabetes [75]. Similarly, *MCP-1* G-2518 gene variant was decreased the risk of type 2 diabetes in a Chinese and Turkey populations [76,77]. These results support a role for CCL2/MCP-1 in pathologies associated with hyperinsulinaemia, although there are contradictory results [78,79].

Besides CCL2/MCP-1, several other chemokines such as CCL5, C-X-C motif chemokine ligand 5 (CXCL5) and CXCL14 are also involved in adipose tissue macrophage infiltration and obesity-induced insulin resistance [46,80,81]. High levels of multiple chemokine ligands (*CCL2*, *CCL3*, *CCL5*, *CCL7*, *CCL8*, *CCL11*) and receptors (*CCR1*, *CCR2*, *CCR3*, *CCR5*) have been observed in adipose tissue of obese subjects and are associated with increased inflammation [69]. Similarly, Tourniaire *et al.* [82] have reported that expression of numerous chemokines (*CCL2*, *CCL5*, *CCL7*, *CCL19*, *CXCL1*, *CXCL5*, *CXCL8*, *CXCL10*) is increased in adipose tissue of obese subjects compared to lean subjects. Thus, these studies suggest possibility that loss of one chemokine may be compensated by other chemokines.

### 4.2. TNF-α

TNF-α is a pro-inflammatory cytokine that may contribute to the pathogenesis of obesity and insulin resistance [36]. Expression of TNF-α is increased in obesity and insulin resistance in humans and is positively correlated with insulin resistance [36]. Treatment with TNF-α induces insulin resistance in adipose tissue [83], whereas deletion of *TNF-α* or its receptors improves insulin sensitivity in obese animals [84]. However, the correlation between plasma TNF-α levels and insulin resistance is relatively weak [36,85], and chronic neutralization of TNF-α does not improve insulin resistance in healthy overweight subjects with metabolic syndrome and insulin resistance, despite improvements in inflammatory status [86]. Bernstein *et al.* [87] have also reported that administration of TNF-α antagonist does not improve insulin sensitivity in humans. The absence of an effect on insulin sensitivity may be due to a compensatory role of other cytokines in the absence of TNF-α, since metabolic dysregulation has been attributed to numerous pro-inflammatory cytokines secreted by adipose tissue, including TNF-α, IL-1, and IL-6, all of which have been involved in disrupting insulin signaling [88]. TNF-α is a part of complex inflammation network and is capable of initiating cytokine cascades involving both synergistic and inhibitory reactions, which control the synthesis and expression of other cytokines, hormones, and their receptors [89]. For example, In *TNF-α* null mice, serum IL-12 levels were increased [90]. Because IL-12 and TNF-α are co-stimulators for IFN-α, one of the essential cytokines for regulation of the inflammation and insulin resistance in obesity [91], the up-regulation of IL-12 in the absence of TNF-α could act in a compensatory manner to induce and maintain appropriate IFN-α levels. In addition, TNF-α does not induce insulin resistance when *IL-6* is down-regulated in adipose tissue [92].

### 4.3. IL-6 and IL-18

IL-6 is another cytokine that plays an important role in the development of insulin resistance in obesity [93]. Adipose tissue contributes to 10%–35% of circulating IL-6 levels in humans [94], and hypertrophic enlargement of adipocytes is accompanied by increased production of IL-6 by adipose tissue [95]. Expression of adipose IL-6 positively correlates with insulin resistance both *in vivo* and *in vitro* [96]. Hyperglycemia results in increased IL-6 levels [97], and treatment with IL-6 induces hyperglycemia and insulin resistance in humans [98]. However, the correlation between IL-6 and obesity or insulin resistance is controversial. A lack of *IL-6* has been shown to cause obesity and insulin resistance in mice [99], but Di Gregorio *et al.* [100] did not observe any obvious phenotype related to obesity and diabetes in *IL-6*-deficient mice compared with wild-type mice. IL-6 appears to have different actions depending on the tissue (*i.e.*, skeletal muscle *vs*. adipose tissue). Treatment of IL-6 enhances insulin-stimulated glucose disposal in humans *in vivo*, and it increases glucose uptake and fatty acid oxidation in cultured L6 myotubes via activation of adenosine monophosphate-activated protein kinase (AMPK), as well as having an anti-inflammatory effect [101,102], whereas IL-6 induces insulin resistance in adipocytes [39]. Thus, the different tissue-specific functions of IL-6 may account for the controversial findings regarding the correlation between IL-6 and insulin resistance.

IL-18 is also a pro-inflammatory cytokine and has been suggested to be produced by adipose tissue [103]. Circulating IL-18 levels have been shown to be increased in obese subjects and reduced with weight loss [104]. Moreover, overexpression of *IL-18* aggravated insulin resistance in a rat model of metabolic syndrome [105]. However, a lack of *IL-18* or its receptor in mice induces hyperphagia, obesity and insulin resistance [106]. Thus, further studies are needed to evaluate the role of IL-6 and IL-18 in the pathogenesis of obesity and insulin resistance.

### 4.4. Leptin

Leptin is abundantly expressed in adipose tissue, specifically adipocytes and is involved in the regulation of energy homeostasis [107]. It inhibits appetite and food intake and stimulates energy expenditure [107]. However, circulating leptin levels [108] and its mRNA expression in adipose tissue [109] are increased in obese subjects, probably due to the existence of leptin resistance [107]. Leptin also plays an important role in the regulation of glucose homeostasis, independent of actions on food intake, energy expenditure or body weight. Leptin improves insulin sensitivity in the liver and skeletal muscle and regulates pancreatic β-cell function [110], whereas it impairs insulin signaling in murine adipocytes [111,112]. In addition, leptin is suggested to have pro-inflammatory effects; Leptin has a cytokine-like structure, and its receptor is member of the class I cytokine receptor (gp130) superfamily [113]. It not only promotes the production of the pro-inflammatory cytokines, IL-2 and IFN-γ, but also inhibits the production of the anti-inflammatory cytokine IL-4 by T cells or mononuclear cells [114]. Concomitantly, circulating leptin levels and its expression in adipose tissues are increased in response to pro-inflammatory cytokines (TNF, IL-1) and endotoxin (lipopolysaccharide, LPS) [115]. Accordingly, the interactions between leptin and inflammation are bidirectional: Pro-inflammatory cytokines increase the synthesis and release of leptin, which in turn contribute to maintain a chronic inflammatory state in obesity [113].

### 4.5. Resistin

Resistin is also an adipocyte-specific secreted adipokine, and it promotes both inflammation and insulin resistance in murine models. Levels of circulating resistin are increased in obese mice and correlated with insulin resistance [116,117], whereas a lack of resistin protects mice from diet-induced hyperglycemia by increasing the activity of AMPK and decreasing the expression of gluconeogenic enzymes in the liver [118]. Moreover, resistin inhibits multiple steps involved in insulin signaling in 3T3-L1 adipocytes and induces the expression of suppressor of *cytokine signaling-3* (*SOCS-3*), a known inhibitor of insulin signaling, in both 3T3-L1 adipocytes and murine adipose tissues [119]. However, there are conflicting reports of the potency of resistin in metabolic diseases in humans. Several studies have consistently reported a close relationship between resistin levels and obesity, insulin resistance, or type 2 diabetes [120–124]. However, other studies have shown that circulating resistin levels and adipocyte expression are not associated with insulin resistance in humans [125,126]. Unlike mouse resistin, human *resistin* is exclusively expressed in mononuclear cells including macrophages [126], and macrophage-derived human resistin exacerbates adipose tissue inflammation and insulin resistance in mice [127].

### 4.6. PAI-1

PAI-1, a primary inhibitor of fibrinolysis, is also synthesized by adipocytes as well as stromal vascular cells, such as preadipocytes, fibroblasts, vascular endothelial cells, and a variety of immune cells, in adipose tissue, and its levels in plasma are increased in obesity and insulin resistance [128,129]. A deficiency of *PAI-1* decreases body weight gain, increases total energy expenditure, and improves insulin resistance in mice fed a high-fat diet [130]. Moreover, mice lacking *PAI-1* have promoted adipocyte differentiation and enhanced basal glucose uptake as well as insulin-stimulated glucose uptake [131]. PAI-1 regulates expression of inflammatory factors, such as IL-8 and leukotriene B4, and monocyte migration, and its expression is regulated by various cytokine inducers such as cigarette smoke extraction and LPS [132].

### 4.7. Visfatin

Visfatin, which was previously identified as a modulator of β-cell differentiation that is expressed in a variety of tissues and cell types, including lymphocytes, bone marrow, muscle and liver [133], has been reported to be secreted by adipose tissue, especially visceral adipose tissue, and exhibit insulin-like activities in mice [134,135]. *Visfatin* was highly expressed in the visceral adipose tissue of mice as well as humans, and treatment with visfatin enhances glucose uptake in adipocytes and myocytes [134]. However, several studies have failed to confirm that visfatin is expressed predominantly in visceral white adipose tissue [136–139] and that expression of *visfatin* in adipose tissue is related to obesity [136,139,140]. Moreover, several studies have reported that circulating visfatin levels are high in subjects with obesity and type 2 diabetes and are positively associated with insulin resistance [139–141]. However, serum visfatin levels and expression of *visfatin* in adipose tissue are not correlated with glucose metabolism or insulin resistance [137,142]. A recent study has demonstrated that central visfatin improves hypothalamic insulin signaling and increases glucose-stimulated insulin secretion and β-cell mass without changing serum visfatin levels in diabetic rats [143]. Oki *et al.* [144] reported that, although not related to insulin resistance in humans, serum visfatin levels are positively correlated with serum levels of IL-6 and *C*-reactive protein, which are known to be pro-inflammatory markers. Therefore, further studies are needed to clarify the role of visfatin in the pathogenesis of obesity induced-insulin resistance.

### 4.8. RBP4

RBP4 is a hepatocyte-synthesized protein that is involved in the transport of vitamin A (retinol) in the body [145]. Recently, it has been suggested that RBP4 is also secreted by adipocytes and affects insulin sensitivity [146]. In states of obesity and insulin resistance, RBP4 is preferentially produced by visceral adipose tissue compared with subcutaneous adipose tissue, and thus it is linked to intra-abdominal adipose tissue expansion [147]. Expression of *RBP4* is increased in adipose tissue in insulin-resistant mice, and adipose tissue *RBP4* mRNA expression is correlated with changes in serum RBP4 levels [147]. In primary human adipocytes, RBP4 inhibits insulin-induced phosphorylation of IRS-1 and ERK1/2, which may be involved in integrating nutrient sensing with insulin signaling [148]. Clinical studies have also reported that circulating RBP4 levels are associated with insulin resistance in subjects with obesity, impaired glucose tolerance, or type 2 diabetes as well as in nonobese subjects [149,150]. Along with markers of obesity and insulin resistance, RBP4 is correlated with inflammatory factors [151]. Several *RBP4* gene variants are associated with adiposity and insulin resistance [152–154]. However, in several clinical studies, circulating RBP4 levels were not associated with obesity and insulin resistance [155,156]. Furthermore, some studies showed no correlation between serum RBP4 levels and expression of *RBP4* in adipose tissue [155,157]. Thus, the relationship between adipose RBP4 expression, circulating levels of RBP4, obesity and insulin resistance in humans needs to be evaluated in future studies.

### 4.9. ANGPTL2

ANGPTL2 was recently identified as an adipocyte-derived inflammatory mediator that promotes inflammation and insulin resistance [158]. Expression of *ANGPTL2* in adipose tissue and circulating levels of ANGPTL2 are higher in diet-induced obese mice than in control mice, and circulating levels of ANGPTL2 are closely related to adiposity, insulin resistance, and inflammation in mice [158]. A deficiency of *ANGPTL2* improves adipose tissue inflammation and insulin resistance in diet-induced obese mice, whereas its overexpression in adipose tissue promotes inflammation as well as insulin resistance in mice [158]. ANGPTL2 is also closely associated with adiposity and inflammation in humans [158]. Recently, Doi *et al.* [159] reported that circulating ANGPTL2 levels are positively correlated with the development of type 2 diabetes in humans, and this relationship is independent of other risk factors for type 2 diabetes, including high-sensitivity *C*-reactive protein levels. Further studies are needed to identify the association of human adipose ANGPTL2 expression with the development of type 2 diabetes.

### 4.10. Adiponectin

Adiponectin is a well-known adipose-specific adipokine that produces insulin-sensitizing effects. Levels of adiponectin are low in obese subjects, and treatment with adiponectin increases insulin sensitivity in animal models [160,161]. Expression of *adiponectin* in adipose tissue is lower in subjects with obesity and insulin resistance than in lean subjects and is associated with higher degrees of insulin sensitivity and lower adipose *TNF-α* expression [162]. A deficiency of *adiponectin* in mice induces insulin resistance, whereas over-expression of *adiponectin* in mice improves insulin sensitivity and glucose tolerance [163]. It has been reported that the adiponectin receptors, *adiponectin receptor* (*AdipoR*)*1* and *AdipoR2* are reduced in obesity-related insulin resistance and mediate the anti-metabolic actions of adiponectin [164].

### 4.11. SFRP5

SFRP5 is a new adipokine with insulin sensitizing and anti-inflammatory properties that exhibits beneficial effects on metabolic dysfunction [165]. The SFRP5 gene and protein are expressed at higher levels in adipose tissue than in other tissues and, in particular, gene expression is confined to adipocytes rather than stromal vascular cells [165]. A deficiency of *SFRP5* in mice induced impaired insulin sensitivity, increased risk of developing NAFLD and aggravated adipose inflammation compared with control mice when fed a high-calorie diet, although *SFRP5*-deficient mice did not show detectable phenotype changes on a regular diet [165]. Conversely, administration of SFRP5 improves metabolic function and reduces adipose inflammation in obese and diabetic mice. The metabolic dysfunction observed in *SFRP5*-deficient mice is associated with increased accumulation of macrophages and enhanced production of pro-inflammatory cytokines in adipose tissue [165]. Furthermore, in *SFRP5*-deficient mice, *JNK1* loss reverses the impaired insulin sensitivity and increased adipose inflammation [165], suggesting that a deficiency of *SFRP5* promotes obesity-induced inflammation and metabolic dysfunction via activation of JNK1 in adipose tissue. Clinical study also demonstrated that plasma levels of SFRP5 were lower in adult subjects with impaired glucose intolerance and type 2 diabetes than normal glucose tolerance subjects, and its levels were negatively correlated with body mass index (BMI), waist-to-hip ratio and HOMA-IR [166,167]. In addition, circulating SFRP5 was associated with obesity and metabolic syndrome in obese children, and its levels were increased after weight loss [168]. However, Carstensen *et al.* [169] reported a positive correlation between serum SFRP5 levels and parameters of glucose homeostasis or insulin resistance in healthy and obese subjects. Future clinical studies are required to determine the role of adipose SFRP5 in the control of obesity-related abnormalities in glucose homeostasis and insulin sensitivity.

## 5. Obesity and Dyslipidemia

**Figure 4.**
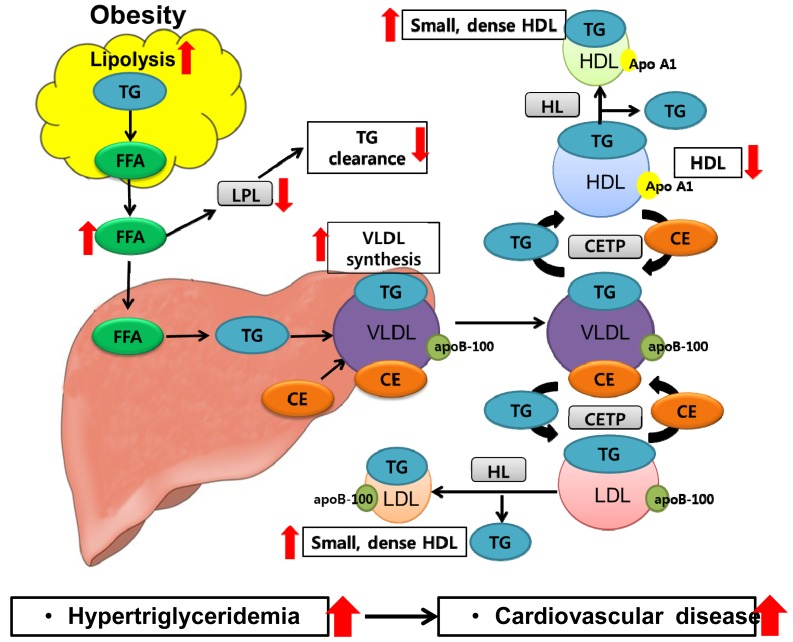
Mechanisms of dyslipidemia in obesity. An increased free fatty acids (FFA) release from adipose tissue via lipolysis can result in enhanced delivery of FFA to the liver. The enhanced FFA leads to increased triglyceride (TG) and very-low-density lipoprotein (VLDL) production in the liver as well as inhibition of lipoprotein lipase in adipose tissue and skeletal muscle, thereby promoting hypertriglyceridemia. Moreover, the increased VLDL in the liver can inhibit lipolysis of chylomicrons, which also contributes to hypertriglyceridemia. The TG in VLDL is exchanged for cholesteryl esters from low-density lipoproteins (LDL) and high-density lipoproteins (HDL) by the cholesteryl ester transport protein, producing TG-rich LDL and HDL. The TG in the LDL and HDL is then hydrolyzed by hepatic lipase, producing both small, dense LDL and HDL. The decreased HDL concentration and formation of small, dense LDL particules are linked to a higher risk of cardiovascular disease. The red arrows indicate increased (when pointing upward) or decreased (when pointing downward) responses to obesity. CE, cholesteryl esters; CETP, cholesteryl ester transport protein; FFA, free fatty acids; HDL, high-density lipoproteins; HL, hepatic lipase; LDL, low-density lipoproteins; LPL, lipoprotein lipase; TG, triglyceride; VLDL, very-low-density lipoprotein.

Obesity is also linked to an increased prevalence of dyslipidemia. Dyslipidemia is an abnormal amount of lipids, such as cholesterol and triglyceride, in the blood and is a widely accepted risk factor for cardiovascular disease. Obesity-related dyslipidemia is primarily characterized by increased levels of plasma free fatty acids and triglycerides, decreased levels of high-density lipoprotein (HDL), and abnormal low-density lipoprotein (LDL) composition (Figure 4). The most significant contributing factor for obesity-related dyslipidemia is likely uncontrolled fatty acid release from adipose tissue, especially visceral adipose tissue, through lipolysis, which causes increased delivery of fatty acids to the liver and synthesis of very-low-density lipoprotein (VLDL). Increased levels of free fatty acids can decrease mRNA expression or activity of lipoprotein lipase (LPL) in adipose tissue and skeletal muscle, and increased synthesis of VLDL in the liver can inhibit lipolysis of chylomicrons, which promotes hypertriglyceridemia [170–172]. Hypertriglyceridemia further triggers a cholesterylester transfer protein-mediated exchange of triglycerides for cholesterol esters between triglyceride-rich lipoproteins (VLDL, immediate-density lipoprotein) and lipoproteins, which are relatively richer in cholesterol esters (LDL, HDL), which leads to a decreased HDL-cholesterol concentration and a reduction in triglyceride content in LDL [173]. The increased triglyceride content in LDL is hydrolyzed by hepatic lipase (HL) [173], leading to the formation of small, dense LDL particles that are associated with a higher risk of cardiovascular disease [174]. For decades, in clinical practice, LDL cholesterol has been the cornerstone measurement for assessing cardiovascular risk and is typically estimated using Friedewald formula [175]. As the formula is calculated based on measurements of total cholesterol, triglyceride, HDL cholesterol, the accuracy of Friedewald formula depends on the accuracy of these values. Therefore, recently, the limitation and errors of the Friedewald equation are not well appreciated by clinicians although well-documented. Currently, there are several homogeneous assays for LDL cholesterol based on selective detergents or other elimination methods to separate chylomicrons, VLDL, and HDL from LDL [176]. A homogeneous assay for measurement of small, dense LDL cholesterol has also been developed [177]. The selective measurement of the small, dense LDL cholesterol concentration is crucial for evaluating the actual atherogenic risk of individuals, since a high concentration of small, dense LDL cholesterol is closely related to a high prevalence of cardiovascular disease [178]. Rizzo *et al.* [179] suggested the predictive role of small, dense LDL beyond traditional cardiovascular risk factors in subjects with metabolic syndrome events, and National Cholesterol Education Program-Adult Treatment Panel III has accepted the sd-LDL as a novel cardiovascular risk factor.

Adipocyte size is suggested to be an important factor for determining the degree to which adipose tissue contributes to dyslipidemia. Enlargement of adipocytes is associated with an increase in lipolysis [180], which leads to further increases in levels of circulating free fatty acids and their delivery to the liver to increase triglyceride synthesis. Along with triglyceride synthesis in the liver, the increased delivery of free fatty acids to the liver exacerbates insulin resistance, which promotes dyslipidemia. Obese subjects have higher whole body fatty acid release compared with lean subjects because of their greater fat mass, although their basal adipose tissue lipolysis per kilogram of fat is lower [13]. A recent study reported the association between enlargement of visceral adipocytes, but not subcutaneous adipocytes, and dyslipidemia independent of body composition and fat distribution in obese subjects [181]. A relationship between visceral adipose tissue and dyslipidemia was also found in patients with type 2 diabetes [182]. The content of visceral adipose tissue is positively correlated with the number of VLDLs and LDLs, even when controlling for BMI and distribution of subcutaneous adipose tissue [182]. Expansion of visceral adipose tissue has also been associated with larger VLDL particles as well as smaller LDL and HDL particles, which have a lower capacity to transfer cholesteryl esters in reverse cholesterol transport and predict atherosclerosis [182]. Visceral adipose tissue has higher lipolytic rates than subcutaneous adipose tissue, and free fatty acids are directly delivered to the liver through the portal vein [183]. Independent of total body fat, the expanded visceral adipose tissue is positively correlated with high hepatic triglyceride lipase activity [184]. A high amount of visceral adipose tissue is also positively correlated with increased HL activity [185] which is associated with increased cardiovascular risk [174].

Many adipose-produced inflammatory molecules, including TNF-α, IL-6, IL-1, serum amyloid A (SAA) and adiponectin, and the number of adipose macrophages also play an important role in the development of dyslipidemia. As noted in the preceding text, obese subjects have higher levels of macrophage infiltration into adipose tissue compared with lean controls, leading to the increased levels of pro-inflammatory cytokines and circulating free fatty acids that are involved in the pathogenesis of dyslipidemia. Macrophage infiltration into visceral adipose tissue is positively correlated with circulating triglyceride levels in obese patients, and a negative relationship has been found with plasma HDL cholesterol levels [186]. Moreover, in subcutaneous adipose tissue, a macrophage-specific marker (*CD68*) is positively correlated with levels of plasma free fatty acid as well as LDL and negatively correlated with HDL levels [69]. In addition, inflammation can modify the size, composition and function of HDLs, which leads to the impairment of reverse cholesterol transport and parallel changes in apolipoproteins, cholesterol metabolism-related enzymes, anti-oxidant capacity, and adenosine triphosphate binding cassette A1-dependent efflux [187]. Several adipokines also stimulate lipolysis in adipocytes [188,189] and reduce the clearance of triglyceride-rich particles [190–192]. For example, IL-6 and TNF-α enhanced lipolysis and suppressed activity of LPL, a key regulatory enzyme in the catabolism and clearance of triglyceride-rich lipoproteins, in adipocytes [188,189,191,192].

## 6. Role of Adipose Tissue-Produced Adipokines in Dyslipidemia

### 6.1. Cytokines

TNF-α was originally identified as a factor that induces hypertriglyceridemia in bacteria infected-animals [193]. Levels of plasma TNF-α are higher in hyperlipidemic patients compared with healthy controls and are positively correlated with concentrations of VLDL triglyceride [194]. These effects are related to the promotion of hepatic triglyceride synthesis and secretion [195] as well as inhibition of LPL [196]. In addition, TNF-α directly promotes the overproduction of hepatic apolipoprotein (apo) B100-containing VLDL through impairment of hepatic insulin signaling in animals [197]. Like TNF-α, IL-6 is also associated with hypertriglyceridemia. Subjects with hypertriglyceridemia have a higher production capacity of IL-6 as well as TNF-α [198,199], and increased levels of serum triglycerides are associated with increased levels of IL-6 [200]. Conversely, when anti-inflammatory cytokine (IL-10) levels are increased, plasma triglyceride levels are also increased [201]. Along with TNF-α, pro-inflammatory cytokines, such as IL-6, IL-1, IFN-α and IFN-γ, stimulate triglyceride synthesis in HepG2 cells [202] and/or promote lipolysis in adipocytes [188,189]. In addition, IL-1, IL-6 and IFN-α as well as TNF-α reduce LPL activity *in vivo* and *in vitro* [190–192].

Levels of serum pro-inflammatory cytokines, including TNF-α and IL-6, are negatively correlated with serum HDL-cholesterol levels in healthy subjects and patients with cardiovascular disease [203–205], whereas a positive correlation exists between the anti-inflammatory cytokine (IL-10) concentration and plasma HDL-cholesterol levels [201]. The administration of pro-inflammatory cytokines, such as TNF-α, IL-6 and IL-1, also reduces expression of *apo A1* in hepatic cells and plasma in animals [190,206]. Apo A1 is the major protein component of HDL in plasma, and low concentrations of apoA1 are independent predictors for presence and severity of cardiovascular disease [207].

In contrast to HDL cholesterol, pro-inflammatory cytokines, including TNF-α, IL-6 and IL-1, increase circulating total cholesterol and LDL-cholesterol levels in animals by activating cholesterol synthesis [190,208–210], whereas increased IL-10 levels are negatively associated with high levels of total cholesterol and LDL cholesterol [201]. Pro-inflammatory cytokines (TNF-α, transforming growth factor-β or IL-1) not only promote lipoprotein uptake by scavenger receptor and LDL receptor but also inhibit adenosine triphosphate binding cassette transporter A1 (ABCA1)-mediated cholesterol efflux to HDL, which may contribute to lipid deposition and foam cell formation [211–213]. In addition, TNF-α increases secretion of apo B in rat hepatocyte cultures in the absence of extracellular fatty acids [214]. The level of circulating apo B has been reported to be a strong predictor of coronary artery disease compared with circulating HDL-cholesterol levels [215]. Moreover, in HepG2 cells, the treatment of cytokines (TNF-α, IL-6 and IL-1) stimulates the hepatic production and secretion of phospholipase A2 [216] which accelerate the development of atherosclerosis [217].

### 6.2. SAA

SAA is an apolipoprotein that can replace apo A1 as the major apolipoprotein of HDL [218]. SAA is found in the adipose tissue and the liver in humans [34,219], and mice mainly express it in adipocytes [220]. Expression of *SAA* in adipose tissue and circulating levels of SAA are higher in obese subjects than in lean subjects and are decreased by caloric restriction [219]. A number of studies have suggested a role of SAA in the inflammatory process [221,222]. Treatment with SAA increases expression of the pro-inflammatory cytokines IL-6 and TNF-α in preadipocytes and adipocytes *in vitro* [221,222]. In addition, SAA affects the metabolism of HDL cholesterol through its inhibitory effects on HDL binding and selective lipid uptake mediated by scavenger receptor SR-BI [223], an HDL receptor that mediates the cellular uptake of cholesteryl esters from HDL, thereby promoting the process of reverse cholesterol transport from the periphery to the liver [224]. Lewis *et al*. [225] suggested that SAA might be a potential contributor to atherosclerosis directly by mediating retention of SAA-enriched HDL to vascular proteoglycans, independent of an adverse effect on plasma lipoproteins. Thus, increased expression of SAA can promote dyslipidemia by affecting the HDL structure and function as well as inflammation.

### 6.3. Adiponectin

Adiponectin has beneficial effects on lipid metabolism and also plays a role as a vasoprotective adipokine [226]. Levels of plasma adiponectin have been negatively correlated with triglycerides and positively correlated with HDL cholesterol [71]. Decreased adiponectin levels are associated with dyslipidemia and cardiovascular disease compared with matched controls [227,228]. Adiponectin stimulates fatty acid oxidation and glucose utilization through activation of AMPK in the liver and skeletal muscle, which has been associated with many of the positive effects of adiponectin on lipoprotein metabolism as well as insulin sensitivity [229]. Adiponectin also induces activation of LPL, thereby enhancing VLDL clearance and reducing plasma triglyceride levels [230]. In addition, in subjects with type 2 diabetes and normal controls, low levels of adiponectin have been related to increased HL activity, which may be responsible for the decreased levels of HDL cholesterol [231]. More recently, Matsuura *et al*. [232] reported that adiponectin increases the mRNA expression and secretion of apo A1 as well as ABCA1 mRNA and protein expression in HepG2 cells, suggesting that adiponectin might increase HDL assembly in the liver. In *adiponectin*-knockout mice, plasma and hepatic apo A1 protein levels and hepatic ABCA1 gene and protein expression were shown to be decreased compared with wild-type-mice [233]. Recently, Chang *et al.* [234] suggested that hypoadiponectinemia may be a useful marker of dyslipidemia in subjects with polycystic ovarian syndrome, who have an increased risk of dyslipidemia.

## 7. Obesity and NAFLD

**Figure 5.**
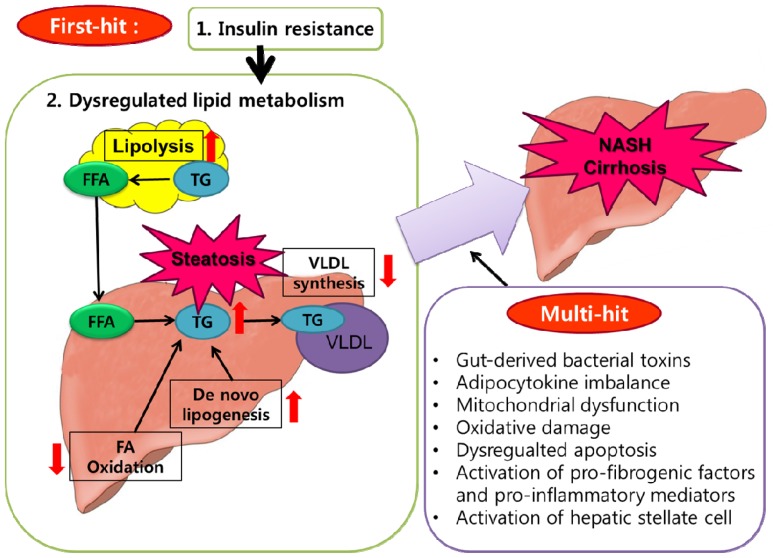
The Multi-hit hypothesis of NAFLD pathogenesis. The “first hit”, such as insulin resistance and lipid metabolism dysregulation, leads to the development of simple steatosis and renders hepatocytes susceptible to “multi-hit”, which include gut-derived bacterial toxins, adipocytokine imbalance, mitochondrial dysfunction, oxidative damage, dysregulated hepatocyte apoptosis, activation of pro-fibrogenic factors and pro-inflammatory mediators and hepatic stellate cell activation, ultimately leading to NASH and cirrhosis. The red arrows indicate up-regulation (when pointing upward) or down-regulation (when pointing downward) in response to insulin resistance.

NAFLD is currently the most common form of chronic liver disease [235], and its incidence has increased in parallel to the rise in the incidence of obesity [3,236]. More than two-thirds of patients with NAFLD are obese [237]. NAFLD is characterized by two steps of liver injury: (1) accumulation of triglycerides in the liver (hepatic steatosis) and (2) inflammation and subsequent fibrosis (nonalcoholic steatohepatitis, NASH) [238]. The “two-hit” hypothesis is widely accepted to explain the development of NAFLD and the progression from simple steatosis to NASH [239]. The “first hit” is the accumulation of hepatic lipids, and the “second hit” promotes hepatocyte injury, inflammation and fibrosis. A number of factors, including proinflammatory cytokines, adipokines, mitochondrial dysfunction, oxidative stress and subsequent lipid peroxidation, initiate the second hit [239]. The classical “two-hit” hypothesis has now been modified by “multi-hit” hypothesis due to involvement of complex factors and interactions leading from lipid dysregulation, adipokine imbalance, adipose inflammation, oxidative stress, insulin resistance to NAFLD [240,241] (Figure 5). In the “multi-hit” hypothesis, imbalanced lipid metabolism and insulin resistance is considered as the “first hit”. Hyperinsulinemia, caused by insulin resistance, results in steatosis via increased *de novo* hepatic lipogenesis, decreased free fatty acid oxidation, decreased hepatic VLDL secretion and increased efflux of free fatty acids due to increased lipolysis from adipose tissue. After the development of steatosis, liver becomes more vulnerable to “multi-hit” including the gut-derived bacterial toxins, adipokine/cytokine imbalance, mitochondrial dysfunction, oxidative damage, dysregulated hepatocyte apoptosis, release of pro-fibrogenic factors and pro-inflammatory mediators from impaired organelles and activation of hepatic stellate cell and Kupffer cell. Such multiple factors may collectively stimulate inflammation, apoptosis and fibrosis that ultimately leading to progressive liver disease.

Excessive lipid accumulation in the liver generally occurs when the influx of lipids, via increased fatty acid import or de novo fatty acid synthesis, exceeds the ability of hepatic lipid clearance by fatty acid oxidation or triglyceride export [242,243]. Recent study has confirmed that increased *de novo* lipogenesis is a distinct characteristic of subjects with NAFLD [244]. As described in the preceding text, adipose tissue is suggested to be a source of free fatty acids and other factors entering the portal circulation [183,245]. Expanded adipose tissue promotes macrophage infiltration and secretion of many pro-inflammatory chemokines, cytokines and adipokines that are closely related to insulin resistance [5,6,40]. A failure to suppress lipolysis by insulin then results in increased release of free fatty acids from adipose tissue [246,247]. The increased lipolysis in adipose tissue, especially visceral adipose tissue [13,180,248], increases free fatty acid influx directly into the liver by the portal vein [246]. The free fatty acids from enlarged adipose tissue are then taken up by the hepatocytes, which lead to reduced hepatic insulin clearance with a further increase in circulating insulin levels [53]. In the liver, free fatty acids promote increased glucose production and triglyceride synthesis and impair insulin suppression of hepatic glucose output [53]. In addition, free fatty acids are ligands of the membrane-bound TLR4 and can promote inflammation [24,28]. However, it is still unclear to what extent the portally drained adipose tissue influences hepatic steatosis. The contribution of visceral adipose tissue lipolysis to the delivery of hepatic free fatty acids has been shown to be only 5%–10% in normal-weight subjects and up to 25% in intra-abdominally obese subjects [245]; however, in the fasting state, hepatic fatty acids originate predominantly from circulating free fatty acids [249] and delivery of the portal free fatty acids to the liver is increased postprandially [250]. Nevertheless, increased free fatty acid influx is a key contributor to promoting accumulation of lipids in the liver, irrespective of the origin of the free fatty acids [247,251].

In the obese state, pro-inflammatory and anti-inflammatory factors secreted by inflamed adipose tissue are also associated with NAFLD [247]. Among them, adiponectin is suggested to protect the liver from steatosis and inflammation. In the liver, adiponectin increases the ability of insulin to suppress glucose production and glucose output [160]. Moreover, it inhibits hepatic lipogenesis by down-regulating the lipogenic transcription factor, *SREBP1-c* [252] and promotes glucose utilization and fatty-acid oxidation in the liver by activating AMPK [229]. These findings are supported by studies in which recombinant adiponectin given to obese mice not only ameliorated hepatomegaly, hepatic steatosis and inflammation but also normalized levels of alanine aminotransferase (ALT) [253] which is a sensitive indicator of liver injury and often used as a surrogate marker for NAFLD [254]. In addition to its metabolic effects, adiponectin has anti-inflammatory activities that might protect the progression of hepatic steatosis to fibrosis. In KK-Ay obese mice, adiponectin attenuated LPS-induced liver injury by decreasing TNF-α levels and activating *peroxisome proliferator-activated receptor* (*PPAR*)*α* in the liver [255]. Moreover, liver fibrosis induced by the administration of carbon tetrachloride was enhanced in *adiponectin*-deficient mice, whereas injection of adiponectin attenuated liver fibrosis in wild-type mice treated with carbon tetrachloride [256]. In accordance with animal studies, a number of clinical studies have suggested a protective role of adiponectin in NAFLD. Circulating adiponectin levels are lower in subjects with NAFLD than in healthy controls [257] and negatively correlated with liver function markers in healthy subjects [258]. Similarly, low adiponectin levels predict hepatic steatosis and increased liver injury enzyme levels in obese subjects [259]. In addition, expression of *adiponectin* and its receptor (*AdipoR2*) is significantly reduced in the liver of patients with NASH compared with those with simple steatosis [260]. Polymorphisms in the gene encoding *AdipoR1* are also associated with hepatic steatosis in human [261].

Leptin is regarded as another key regulator of NAFLD. It directly stimulates AMPK which is involved in activation of lipid oxidation, such as β-oxidation and glycolysis, as well as inhibition of lipogenesis [262]. Expression of *SREBP1-c* is increased in the liver of leptin-unresponsive fa/fa Zucker diabetic fatty rats [263], and infusion of adenovirus-*leptin* not only decreases hepatic triglyceride synthesis but also increases β-oxidation through down-regulation of *SREBP1-c* and up-regulation of *PPARα* [264]. Moreover, a negative correlation between serum leptin levels and hepatic injury has been observed in humans [265]. Conversely, several clinical studies have reported that the concentration of circulating leptin is positively correlated with high serum ALT or hepatic steatosis, independent of BMI and body fat mass [266,267]. Leptin also increases hepatic fibrosis, whereas a deficiency of *leptin* is related to the decreased hepatic injury in animal models [268]. Leptin enhances the expression of pro-fibrogenic cytokine (transforming growth factor-β1) in Kupffer cells [269,270] and has a direct action on hepatic fibrogenesis by activating hepatic stellate cells and stimulating production of α-smooth muscle actin, collagen and tissue inhibitor of metalloproteinase 1 [267,269]. Leptin has been reported to be a potent hepatic stellate cell mitogen and inhibit hepatic stellate cell apoptosis, which promotes the pathogenesis of liver fibrosis [270].

In animal models, resistin also regulates glucose and lipid metabolism in the liver and acts as a mediator of hepatic insulin resistance. Circulating levels of resistin are increased in patients with NAFLD [271,272]. When NAFLD patients is divided according to liver histology (pure fatty liver *vs.* NASH), serum resistin levels are higher in patients with NASH than in those with pure fatty liver and positively correlated with the NASH score, an index that takes into account necrosis, inflammation, and fibrosis in liver biopsies and reflects the severity of the disease. However, the role of resistin in humans remains uncertain. In addition, TNF-α mediates not only the early stages of NAFLD but also the transition to more advanced stages of liver damage in animals and human, suggesting TNF-α has been proposed to play a key role in the development of NASH/NAFLD [273–275]. Moreover, IL-6 and TNF-α increase expression of SOCS in the liver which is involved in increased hepatic SREBP-1c expression and insulin resistance [276]. Acylation-stimulating protein and angiotensinogen have also been observed in adipose tissue, and angiotensinogen levels are increased in obese subjects [277–279]. The levels of acylation-stimulating protein correlate with insulin resistance in NAFLD [277], and angiotensin II antagonists have been shown to improve liver function test results in patients with NAFLD and attenuated fibrosis in animal models [280].

## 8. Conclusions

Obesity, especially visceral obesity, is associated with metabolic disturbances, such as insulin resistance, dyslipidemia and NAFLD. Enlarged adipose tissue results in the infiltration of macrophages and unbalance of pro-inflammatory and anti-inflammatory factors secreted by adipose tissue, which lead to the promotion of inflammation, impairment of insulin sensitivity and dysregulation of lipid metabolism. Excess free fatty acids also contribute to the initiation and progression of obesity-induced metabolic complications. The adipose tissue can affect many other tissues, including the liver, skeletal muscle and heart, via the production of free fatty acids and many pro-inflammatory and anti-inflammatory factors, and therefore has a critical role in the pathogenesis of insulin resistance, dyslipidemia and NAFLD. Although the cause-and-effect association has not been definitively established, available evidence have provided great insight into the critical role of adipose tissue in metabolic syndrome. Thus, further elucidation of the functions and mechanisms of adipose tissue-released bioactive substances will lead to a better understanding of the development of obesity-related metabolic syndrome, and it may provide novel therapeutic approaches to prevent or treat obesity and its metabolic complications. In addition, it will be also worthwhile to focus on how each adipokine signaling pathway integrates with multiple intracellular signaling cascades activated by other factors in the adipose tissue and other tissues.

## References

[1] World Health Organization Obesity and Overweight.

[2] Wang Y.C., McPherson K., Marsh T., Gortmaker S.L., Brown M. (2011). Health and economic burden of the projected obesity trends in the USA and the UK. Lancet.

[3] López-Velázquez J.A., Silva-Vidal K.V., Ponciano-Rodríguez G., Chávez-Tapia N.C., Arrese M., Uribe M., Méndez-Sánchez N. (2014). The prevalence of nonalcoholic fatty liver disease in the Americas. Ann. Hepatol.

[4] Carr D.B., Utzschneider K.M., Hull R.L., Kodama K., Retzlaff B.M., Brunzell J.D., Shofer J.B., Fish B.E., Knopp R.H., Kahn S.E. (2004). Intra-abdominal fat is a major determinant of the National Cholesterol Education Program Adult Treatment Panel III criteria for the metabolic syndrome. Diabetes.

[5] Xu H., Barnes G.T., Yang Q., Tan G., Yang D., Chou C.J., Sole J., Nichols A., Ross J.S., Tartaglia L.A. (2003). Chronic inflammation in fat plays a crucial role in the development of obesity-related insulin resistance. J. Clin. Investig.

[6] Hotamisligil G.S. (2006). Inflammation and metabolic disorders. Nature.

[7] Lumeng C.N., Saltiel A.R. (2011). Inflammatory links between obesity and metabolic disease. J. Clin. Investig.

[8] Kershaw E.E., Flier J.S. (2004). Adipose tissue as an endocrine organ. J. Clin. Endocrinol. Metab.

[9] Hauner H. (2005). Secretory factors from human adipose tissue and their functional role. Proc. Nutr. Soc.

[10] Halberg N., Wernstedt-Asterholm I., Scherer P.E. (2008). The adipocyte as an endocrine cell. Endocrinol. Metab. Clin. N. Am.

[11] Perseghin G., Ghosh S., Gerow K., Shulman G.I. (1997). Metabolic defects in lean nondiabetic offspring of NIDDM parents: A cross-sectional study. Diabetes.

[12] Boden G. (2008). Obesity and free fatty acids. Endocrinol. Metab. Clin. N. Am.

[13] Horowitz J.F., Coppack S.W., Paramore D., Cryer P.E., Zhao G., Klein S. (1999). Effect of short-term fasting on lipid kinetics in lean and obese women. Am. J. Physiol.

[14] Large V., Reynisdottir S., Langin D., Fredby K., Klannemark M., Holm C., Arner P. (1999). Decreased expression and function of adipocyte hormone-sensitive lipase in subcutaneous fat cells of obese subjects. J. Lipid Res.

[15] Hellström L., Reynisdottir S. (2000). Influence of heredity for obesity on adipocyte lipolysis in lean and obese subjects. Int. J. Obes. Relat. Metab. Disord.

[16] McQuaid S.E., Hodson L., Neville M.J., Dennis A.L., Cheeseman J., Humphreys S.M., Ruge T., Gilbert M., Fielding B.A., Frayn K.N. (2011). Downregulation of adipose tissue fatty acid trafficking in obesity: A driver for ectopic fat deposition?. Diabetes.

[17] Langin D., Dicker A., Tavernier G., Hoffstedt J., Mairal A., Rydén M., Arner E., Sicard A., Jenkins C.M., Viguerie N. (2005). Adipocyte lipases and defect of lipolysis in human obesity. Diabetes.

[18] Jocken J.W., Langin D., Smit E., Saris W.H., Valle C., Hul G.B., Holm C., Arner P., Blaak E.E. (2007). Adipose triglyceride lipase and hormone-sensitive lipase protein expression is decreased in the obese insulin-resistant state. J. Clin. Endocrinol. Metab.

[19] Karpe F., Dickmann J.R., Frayn K.N. (2011). Fatty acids, obesity, and insulin resistance: Time for a reevaluation. Diabetes.

[20] Hausman D.B., DiGirolamo M., Bartness T.J., Hausman G.J., Martin R.J. (2001). The biology of white adipocyte proliferation. Obes. Rev.

[21] Spalding K.L., Arner E., Westermark P.O., Bernard S., Buchholz B.A., Bergmann O., Blomqvist L., Hoffstedt J., Naslund E., Britton T. (2008). Dynamics of fat cell turnover in humans. Nature.

[22] Tchoukalova Y.D., Votruba S.B., Tchkonia T., Giorgadze N., Kirkland J.L., Jensen M.D. (2010). Regional differences in cellular mechanisms of adipose tissue gain with overfeeding. Proc. Natl. Acad. Sci. USA.

[23] Wang Y., Rimm E.B., Stampfer M.J., Willett W.C., Hu F.B. (2005). Comparison of abdominal adiposity and overall obesity in predicting risk of type 2 diabetes among men. Am. J. Clin. Nutr.

[24] Kim J.Y., van de Wall E., Laplante M., Azzara A., Trujillo M.E., Hofmann S.M., Schraw T., Durand J.L., Li H., Li G. (2007). Obesity-associated improvements in metabolic profile through expansion of adipose tissue. J. Clin. Investig.

[25] Weyer C., Foley J.E., Bogardus C., Tataranni P.A., Pratley R.E. (2000). Enlarged subcutaneous abdominal adipocyte size, but not obesity itself, predicts type II diabetes independent of insulin resistance. Diabetologia.

[26] Boden G. (1997). Role of fatty acids in the pathogenesis of insulin resistance and NIDDM. Diabetes.

[27] Kelley D.E., Mokan M., Simoneau J.A., Mandarino L.J. (1993). Interaction between glucose and free fatty acid metabolism in human skeletal muscle. J. Clin. Investig.

[28] Shi H., Kokoeva M.V., Inouye K., Tzameli I., Yin H., Flier J.S. (2006). TLR4 links innate immunity and fatty acid-induced insulin resistance. J. Clin. Investig.

[29] Suganami T., Nishida J., Ogawa Y. (2005). A paracrine loop between adipocytes and macrophages aggravates inflammatory changes: Role of free fatty acids and tumor necrosis factor alpha. Arterioscler. Thromb. Vasc. Biol.

[30] Byrne C.D., Maison P., Halsall D., Martensz N., Hales C.N., Wareham N.J. (1999). Cross-sectional but not longitudinal associations between non-esterified fatty acid levels and glucose intolerance and other features of the metabolic syndrome. Diabet. Med.

[31] Cho S.J., Jung U.J., Choi M.S. (2012). Differential effects of low-dose resveratrol on adiposity and hepatic steatosis in diet-induced obese mice. Br. J. Nutr.

[32] Charles M.A., Fontbonne A., Thibult N., Claude J.R., Warnet J.M., Rosselin G., Ducimetière P., Eschwège E. (2001). High plasma nonesterified fatty acids are predictive of cancer mortality but not of coronary heart disease mortality: Results from the paris prospective study. Am. J. Epidemiol.

[33] Reeds D.N., Stuart C.A., Perez O., Klein S. (2006). Adipose tissue, hepatic, and skeletal muscle insulin sensitivity in extremely obese subjects with acanthosis nigricans. Metabolism.

[34] Jernas M., Palming J., Sjoholm K., Jennische E., Svensson P.A., Gabrielsson B.G., Levin M., Sjogren A., Rudemo M., Lystig T.C. (2006). Separation of human adipocytes by size: Hypertrophic fat cells display distinct gene expression. FASEB J.

[35] Skurk T., Alberti-Huber C., Herder C., Hauner H. (2007). Relationship between adipocyte size and adipokine expression and secretion. J. Clin. Endocrinol. Metab.

[36] Hotamisligil G.S., Shargill N.S., Spiegelman B.M. (1993). Adipose expression of tumor necrosis factor-α: Direct role in obesity-linked insulin resistance. Science.

[37] Amrani A., Jafarian-Tehrani M., Mormède P., Durant S., Pleau J.M., Haour F., Dardenne M., Homo-Delarche F. (1996). Interleukin-1 effect on glycemia in the non-obese diabetic mouse at the pre-diabetic stage. J. Endocrinol.

[38] Sartipy P., Loskutoff D.J. (2003). Monocyte chemoattractant protein 1 in obesity and insulin resistance. Proc. Natl. Acad. Sci. USA.

[39] Rotter V., Nagaev I., Smith U. (2003). Interleukin-6 (IL-6) induces insulin resistance in 3T3-L1 adipocytes and is, like IL-8 and tumor necrosis factor-alpha, overexpressed in human fat cells from insulin-resistant subjects. J. Biol. Chem.

[40] Weisberg S.P., McCann D., Desai M., Rosenbaum M., Leibel R.L., Ferrante A.W. (2003). Obesity is associated with macrophage accumulation in adipose tissue. J. Clin. Investig.

[41] Lumeng C.N., Bodzin J.L., Saltiel A.R. (2007). Obesity induces a phenotypic switch in adipose tissue macrophage polarization. J. Clin. Investig.

[42] Jiao P., Chen Q., Shah S., Du J., Tao B., Tzameli I., Yan W., Xu H. (2009). Obesity-related upregulation of monocyte chemotactic factors in adipocytes: Involvement of nuclear factor-kappaB and c-Jun NH2-terminal kinase pathways. Diabetes.

[43] Lee Y.H., Thacker R.I., Hall B.E., Kong R., Granneman J.G. (2014). Exploring the activated adipogenic niche: Interactions of macrophages and adipocyte progenitors. Cell Cycle.

[44] Nomiyama T., Perez-Tilve D., Ogawa D., Gizard F., Zhao Y., Heywood E.B., Jones K.L., Kawamori R., Cassis L.A., Tschöp M.H. (2007). Osteopontin mediates obesity-induced adipose tissue macrophage infiltration and insulin resistance in mice. J. Clin. Investig.

[45] Weisberg S.P., Hunter D., Huber R., Lemieux J., Slaymaker S., Vaddi K., Charo I., Leibel R.L., Ferrante A.W. (2006). CCR2 modulates inflammatory and metabolic effects of high-fat feeding. J. Clin. Investig.

[46] Nara N., Nakayama Y., Okamoto S., Tamura H., Kiyono M., Muraoka M., Tanaka K., Taya C., Shitara H., Ishii R. (2007). Disruption of CXC motif chemokine ligand-14 in mice ameliorates obesity-induced insulin resistance. J. Biol. Chem.

[47] Feng B., Jiao P., Nie Y., Kim T., Jun D., van Rooijen N., Yang Z., Xu H. (2011). Clodronate liposomes improve metabolic profile and reduce visceral adipose macrophage content in diet-induced obese mice. PLoS One.

[48] Clément K., Viguerie N., Poitou C., Carette C., Pelloux V., Curat C.A., Sicard A., Rome S., Benis A., Zucker J.D. (2004). Weight loss regulates inflammation-related genes in white adipose tissue of obese subjects. FASEB J.

[49] Cancello R., Henegar C., Viguerie N., Taleb S., Poitou C., Rouault C., Coupaye M., Pelloux V., Hugol D., Bouillot J.L. (2005). Reduction of macrophage infiltration and chemoattractant gene expression changes in white adipose tissue of morbidly obese subjects after surgery-induced weight loss. Diabetes.

[50] Schipper H.S., Prakken B., Kalkhoven E., Boes M. (2012). Adipose tissue-resident immune cells: Key players in immunometabolism. Trends. Endocrinol. Metab.

[51] Arita Y., Kihara S., Ouchi N., Takahashi M., Maeda K., Miyagawa J., Hotta K., Shimomura I., Nakamura T., Miyaoka K. (1999). Paradoxical decrease of an adipose-specific protein, adiponectin, in obesity. Biochem. Biophys. Res. Commun.

[52] Lillioja S., Mott D.M., Spraul M., Ferraro R., Foley J.E., Ravussin E., Knowler W.C., Bennett P.H., Bogardus C. (1993). Insulin resistance and insulin secretory dysfunction as precursors of non-insulin-dependent diabetes mellitus. N. Engl. J. Med.

[53] Kahn B.B., Flier J.S. (2000). Obesity and insulin resistance. J. Clin. Investig.

[54] Schenk S., Saberi M., Olefsky J.M. (2008). Insulin sensitivity: Modulation by nutrients and inflammation. J. Clin. Investig.

[55] Holland W.L., Brozinick J.T., Wang L.P., Hawkins E.D., Sargent K.M., Liu Y., Narra K., Hoehn K.L., Knotts T.A., Siesky A. (2007). Inhibition of ceramide synthesis ameliorates glucocorticoid-, saturated-fat-, and obesity-induced insulin resistance. Cell Metab.

[56] Dressler K.A., Mathias S., Kolesnick R.N. (1992). Tumor necrosis factor-alpha activates the sphingomyelin signal transduction pathway in a cell-free system. Science.

[57] Teruel T., Hernandez R., Lorenzo M. (2001). Ceramide mediates insulin resistance by tumor necrosis factor-alpha in brown adipocytes by maintaining Akt in an inactive dephosphorylated state. Diabetes.

[58] Haus J.M., Kashyap S.R., Kasumov T., Zhang R., Kelly K.R., Defronzo R.A., Kirwan J.P. (2009). Plasma ceramides are elevated in obese subjects with type 2 diabetes and correlate with the severity of insulin resistance. Diabetes.

[59] Holland W.L., Bikman B.T., Wang L.P., Yuguang G., Sargent K.M., Bulchand S., Knotts T.A., Shui G., Clegg D.J., Wenk M.R. (2011). Lipid-induced insulin resistance mediated by the proinflammatory receptor TLR4 requires saturated fatty acid-induced ceramide biosynthesis in mice. J. Clin. Investig.

[60] Horowitz J.F., Klein S. (2000). Whole body and abdominal lipolytic sensitivity to epinephrine is suppressed in upper body obese women. Am. J. Physiol. Endocrinol. Metab.

[61] Bajaj M., Suraamornkul S., Romanelli A., Cline G.W., Mandarino L.J., Shulman G.I., DeFronzo R.A. (2005). Effect of a sustained reduction in plasma free fatty acid concentration on intramuscular long-chain fatty Acyl-CoAs and insulin action in type 2 diabetic patients. Diabetes.

[62] Santomauro A.T., Boden G., Silva M.E., Rocha D.M., Santos R.F., Ursich M.J., Strassmann P.G., Wajchenberg B.L. (1999). Overnight lowering of free fatty acids with Acipimox improves insulin resistance and glucose tolerance in obese diabetic and nondiabetic subjects. Diabetes.

[63] Girousse A., Tavernier G., Valle C., Moro C., Mejhert N., Dinel A.L., Houssier M., Roussel B., Besse-Patin A., Combes M. (2013). Partial inhibition of adipose tissue lipolysis improves glucose metabolism and insulin sensitivity without alteration of fat mass. PLoS Biol.

[64] Kosteli A., Sugaru E., Haemmerle G., Martin J.F., Lei J., Zechner R., Ferrante A.W. (2010). Weight loss and lipolysis promote a dynamic immune response in murine adipose tissue. J. Clin. Investig.

[65] Apovian C.M., Bigornia S., Mott M., Meyers M.R., Ulloor J., Gagua M., McDonnell M., Hess D., Joseph L., Gokce N. (2008). Adipose macrophage infiltration is associated with insulin resistance and vascular endothelial dysfunction in obese subjects. Arterioscler. Thromb. Vasc. Biol.

[66] Anderson E.K., Gutierrez D.A., Hasty A.H. (2010). Adipose tissue recruitment of leukocytes. Curr. Opin. Lipidol.

[67] Fain J.N. (2010). Release of inflammatory mediators by human adipose tissue is enhanced in obesity and primarily by the nonfat cells: A review. Mediat. Inflamm.

[68] Ouchi N., Parker J.L., Lugus J.J., Walsh K. (2011). Adipokines in inflammation and metabolic disease. Nat. Rev. Immunol.

[69] Huber J., Kiefer F.W., Zeyda M., Ludvik B., Silberhumer G.R., Prager G., Zlabinger G.J., Stulnig T.M. (2008). CC chemokine and CC chemokine receptor profiles in visceral and subcutaneous adipose tissue are altered in human obesity. J. Clin. Endocrinol. Metab.

[70] Deshmane S.L., Kremlev S., Amini S., Sawaya B.E. (2009). Monocyte chemoattractant protein-1 (MCP-1): An overview. J. Interferon Cytokine Res.

[71] Kanda H., Tateya S., Tamori Y., Kotani K., Hiasa K., Kitazawa R., Kitazawa S., Miyachi H., Maeda S., Egashira K. (2006). MCP-1 contributes to macrophage infiltration into adipose tissue, insulin resistance, and hepatic steatosis in obesity. J. Clin. Investig.

[72] Kamei N., Tobe K., Suzuki R., Ohsugi M., Watanabe T., Kubota N., Ohtsuka-Kowatari N., Kumagai K., Sakamoto K., Kobayashi M. (2006). Overexpression of monocyte chemoattractant protein-1 in adipose tissues causes macrophage recruitment and insulin resistance. J. Biol. Chem.

[73] Lumeng C.N., DelProposto J.B., Westcott D.J., Saltiel A.R. (2008). Phenotypic switching of adipose tissue macrophages with obesity is generated by spatiotemporal differences in macrophage subtypes. Diabetes.

[74] Meijer K., de Vries M., Al-Lahham S., Bruinenberg M., Weening D., Dijkstra M., Kloosterhuis N., van der Leij R.J., van der Want H., Kroesen B.J. (2011). Human primary adipocytes exhibit immune cell function: Adipocytes prime inflammation independent of macrophages. PLoS One.

[75] Simeoni E., Hoffmann M.M., Winkelmann B.R., Ruiz J., Fleury S., Boehm B.O., März W., Vassalli G. (2004). Association between the A-2518G polymorphism in the monocyte chemoattractant protein-1 gene and insulin resistance and Type 2 diabetes mellitus. Diabetologia.

[76] Karadeniz M., Erdogan M., Cetinkalp S., Berdeli A., Eroglu Z., Ozgen A.G. (2010). Monocyte chemoattractant protein-1 (MCP-1) 2518G/A gene polymorphism in Turkish type 2 diabetes patients with nephropathy. Endocrine.

[77] Jing Y., Zhu D., Bi Y., Yang D., Hu Y., Shen S. (2011). Monocyte chemoattractant protein 1–2518 A/G polymorphism and susceptibility to type 2 diabetes in a Chinese population. Clin. Chim. Acta.

[78] Zietz B., Büchler C., Herfarth H., Müller-Ladner U., Spiegel D., Schölmerich J., Schäffler A. (2005). Caucasian patients with type 2 diabetes mellitus have elevated levels of monocyte chemoattractant protein-1 that are not influenced by the −2518 A→G promoter polymorphism. Diabetes Obes. Metab.

[79] Katakami N., Matsuhisa M., Kaneto H., Matsuoka T.A., Imamura K., Ishibashi F., Kanda T., Kawai K., Osonoi T., Kashiwagi A. (2010). Monocyte chemoattractant protein-1 (MCP-1) gene polymorphism as a potential risk factor for diabetic retinopathy in Japanese patients with type 2 diabetes. Diabetes Res. Clin. Pract.

[80] Keophiphath M., Rouault C., Divoux A., Clement K., Lacasa D. (2009). CCL5 promotes macrophage recruitment and survival in human adipose tissue. Arterioscler. Thromb. Vasc. Biol.

[81] Chavey C., Lazennec G., Lagarrigue S., Clape C., Iankova I., Teyssier J., Annicotte J.S., Schmidt J., Mataki C., Yamamoto H. (2009). CXC ligand 5 is an adipose-tissue derived factor that links obesity to insulin resistance. Cell Metab.

[82] Tourniaire F., Romier-Crouzet B., Lee J.H., Marcotorchino J., Gouranton E., Salles J., Malezet C., Astier J., Darmon P., Blouin E. (2013). Chemokine Expression in Inflamed Adipose Tissue Is Mainly Mediated by NF-κB. PLoS One.

[83] Ruan H., Lodish H.F. (2003). Insulin resistance in adipose tissue: Direct and indirect effects of tumor necrosis factor-α. Cytokine Growth Factor Rev.

[84] Uysal K.T., Wiesbrock S.M., Marino M.W., Hotamisligil G.S. (1997). Protection from obesity-induced insulin resistance in mice lacking TNF-α function. Nature.

[85] Miyazaki Y., Pipek R., Mandarino L.J., DeFronzo R.A. (2003). Tumor necrosis factor α and insulin resistance in obese type 2 diabetic patients. Int. J. Obes. Relat. Metab. Disord.

[86] Wascher T.C., Lindeman J.H., Sourij H., Kooistra T., Pacini G., Roden M. (2011). Chronic TNF-α neutralization does not improve insulin resistance or endothelial function in “healthy” men with metabolic syndrome. Mol. Med.

[87] Bernstein L.E., Berry J., Kim S., Canavan B., Grinspoon S.K. (2006). Effects of etanercept in patients with the metabolic syndrome. Arch. Intern. Med.

[88] McArdle M.A., Finucane O.M., Connaughton R.M., McMorrow A.M., Roche H.M. (2013). Mechanisms of obesity-induced inflammation and insulin resistance: Insights into the emerging role of nutritional strategies. Front. Endocrinol. (Lausanne).

[89] Illei G.G., Lipsky P.E. (2000). Novel, antigen-specific therapeutic approaches to autoimmuneinflammatory diseases. Curr. Opin. Immunol.

[90] Hodge-Dufour J., Marino M.W., Horton M.R., Jungbluth A., Burdick R.D., Strieter R.M., Noble P.W., Hunter C.A., Pure E. (1998). Inhibition of interferon gamma induced interleukin 12 production—A potential mechanism for the anti-inflammatory activities of tumor necrosis factor. Proc. Natl. Acad. Sci. USA.

[91] O’Rourke R.W., White A.E., Metcalf M.D., Winters B.R., Diggs B.S., Zhu X., Marks D.L. (2012). Systemic inflammation and insulin sensitivity in obese IFN-γ knockout mice. Metabolism.

[92] Sultan A., Strodthoff D., Robertson A.K., Paulsson-Berne G., Fauconnier J., Parini P., Rydén M., Thierry-Mieg N., Johansson M.E., Chibalin A.V. (2009). T cell-mediated inflammation in adipose tissue does not cause insulin resistance in hyperlipidemic mice. Circ. Res.

[93] Fernandez-Real J.M., Ricart W. (2003). Insulin resistance and chronic cardiovascular inflammatory syndrome. Endocr. Rev.

[94] Mohamed-Ali V., Goodrick S., Rawesh A., Katz D.R., Miles J.M., Yudkin J.S., Klein S., Coppack S.W. (1997). Subcutaneous adipose tissue releases interleukin-6, but not tumor necrosis factor-alpha *in vivo*. J. Clin. Endocrinol. Metab.

[95] Sopasakis V.R., Sandqvist M., Gustafson B., Hammarstedt A., Schmelz M., Yang X., Jansson P.A., Smith U. (2004). High local concentrations and effects on differentiation implicate interleukin-6 as a paracrine regulator. Obes. Res.

[96] Bastard J.P., Maachi M., van Nhieu J.T., Jardel C., Bruckert E., Grimaldi A., Robert J.J., Capeau J., Hainque B. (2002). Adipose tissue IL-6 content correlates with resistance to insulin activation of glucose uptake both *in vivo* and *in vitro*. J. Clin. Endocrinol. Metab..

[97] Pradhan A.D., Manson J.E., Rifai N., Buring J.E., Ridker P.M. (2001). *C*-reactive protein, interleukin 6, and risk of developing type 2 diabetes mellitus. JAMA.

[98] Tsigos C., Papanicolaou D.A., Kyrou I., Defensor R., Mitsiadis C.S., Chrousos G.P. (1997). Dose-dependent effects of recombinant human interleukin-6 on glucose regulation. J. Clin. Endocrinol. Metab.

[99] Wallenius V., Wallenius K., Ahren B., Rudling M., Carlsten H., Dickson S.L., Ohlsson C., Jansson J.O. (2002). Interleukin-6-deficient mice develop mature-onset obesity. Nat. Med.

[100] Di Gregorio G.B., Hensley L., Lu T., Ranganathan G., Kern P.A. (2004). Lipid and carbohydrate metabolism in mice with a targeted mutation in the IL-6 gene: Absence of development of age-related obesity. Am. J. Physiol. Endocrinol. Metab.

[101] Starkie R., Ostrowski S.R., Jauffred S., Febbraio M., Pedersen B.K. (2003). Exercise and IL-6 infusion inhibit endotoxin-induced TNF-α production in humans. FASEB J.

[102] Carey A.L., Steinberg G.R., Macaulay S.L., Thomas W.G., Holmes A.G., Ramm G., Prelovsek O., Hohnen-Behrens C., Watt M.J., James D.E. (2006). Interleukin-6 increases insulin-stimulated glucose disposal in humans and glucose uptake and fatty acid oxidation *in vitro* via AMP-activated protein kinase. Diabetes.

[103] Wood I.S., Wang B., Jenkins J.R., Trayhurn P. (2005). The pro-inflammatory cytokine IL-18 is expressed in human adipose tissue and strongly upregulated by TNFalpha in human adipocytes. Biochem. Biophys. Res. Commun.

[104] Esposito K., Pontillo A., Ciotola M., di Palo C., Grella E., Nicoletti G., Giugliano D. (2002). Weight loss reduces interleukin-18 levels in obese women. J. Clin. Endocrinol. Metab.

[105] Tan H.W., Liu X., Bi X.P., Xing S.S., Li L., Gong H.P., Zhong M., Wang Z.H., Zhang Y., Zhang W. (2010). IL-18 overexpression promotes vascular inflammation and remodeling in a rat model of metabolic syndrome. Atherosclerosis.

[106] Netea M.G., Joosten L.A., Lewis E., Jensen D.R., Voshol P.J., Kullberg B.J., Tack C.J., van Krieken H., Kim S.H., Stalenhoef A.F. (2006). Deficiency of interleukin-18 in mice leads to hyperphagia, obesity and insulin resistance. Nat. Med.

[107] Friedman J.M., Halaas J.L. (1998). Leptin and the regulation of body weight in mammals. Nature.

[108] Considine R.V., Sinha M.K., Heiman M.L., Kriauciunas A., Stephens T.W., Nyce M.R., Ohannesian J.P., Marco C.C., McKee L.J., Bauer T.L. (1996). Serum immunoreactive-leptin concentrations in normal-weight and obese humans. N. Engl. J. Med.

[109] Kouidhi S., Jarboui S., Clerget Froidevaux M.S., Abid H., Demeneix B., Zaouche A., Benammar Elgaaied A., Guissouma H. (2010). Relationship between subcutaneous adipose tissue expression of leptin and obesity in Tunisian patients. Tunis. Med.

[110] Marroquí L., Gonzalez A., Ñeco P., Caballero-Garrido E., Vieira E., Ripoll C., Nadal A., Quesada I. (2012). Role of leptin in the pancreatic β-cell: Effects and signaling pathways. J. Mol. Endocrinol.

[111] Müller G., Ertl J., Gerl M., Preibisch G. (1997). Leptin impairs metabolic actions of insulin in isolated rat adipocytes. J. Biol. Chem.

[112] Pérez C., Fernández-Galaz C., Fernández-Agulló T., Arribas C., Andrés A., Ros M., Carrascosa J.M. (2004). Leptin impairs insulin signaling in rat adipocytes. Diabetes.

[113] Paz-Filho G., Mastronardi C., Franco C.B., Wang K.B., Wong M.L., Licinio J. (2012). Leptin: Molecular mechanisms, systemic pro-inflammatory effects, and clinical implications. Arq. Bras. Endocrinol. Metabol.

[114] Lord G.M., Matarese G., Howard J.K., Baker R.J., Bloom S.R., Lechler R.I. (1998). Leptin modulates the T-cell immune response and reverses starvation-induced immunosuppression. Nature.

[115] Grunfeld C., Zhao C., Fuller J., Pollack A., Moser A., Friedman J., Feingold K.R. (1996). Endotoxin and cytokines induce expression of leptin, the ob gene product, in hamsters. J. Clin. Investig.

[116] Satoh H., Nguyen M.T., Miles P.D., Imamura T., Usui I., Olefsky J.M. (2004). Adenovirus-mediated chronic “hyper-resistinemia” leads to *in vivo* insulin resistance in normal rats. J. Clin. Investig.

[117] Qi Y., Nie Z., Lee Y.S., Singhal N.S., Scherer P.E., Lazar M.A., Ahima R.S. (2006). Loss of resistin improves glucose homeostasis in leptin deficiency. Diabetes.

[118] Banerjee R.R., Rangwala S.M., Shapiro J.S., Rich A.S., Rhoades B., Qi Y., Wang J., Rajala M.W., Pocai A., Scherer P.E. (2004). Regulation of fasted blood glucose by resistin. Science.

[119] Steppan C.M., Wang J., Whiteman E.L., Birnbaum M.J., Lazar M.A. (2005). Activation of SOCS-3 by resistin. Mol. Cell. Biol.

[120] Vidal-Puig A., O’Rahilly S. (2001). Resistin: A new link between obesity and insulin resistance?. Clin. Endocrinol.

[121] McTernan C.L., McTernan P.G., Harte A.L., Levick P.L., Barnett A.H., Kumar S. (2002). Resistin, central obesity, and type 2 diabetes. Lancet.

[122] McTernan P.G., McTernan C.L., Chetty R., Jenner K., Fisher F.M., Lauer M.N., Crocker J., Barnett A.H., Kumar S. (2002). Increased resistin gene and protein expression in human abdominal adipose tissue. J. Clin. Endocrinol. Metab.

[123] Wang H., Chu W.S., Hemphill C., Elbein S.C. (2002). Human resistin gene: Molecular scanning and evaluation of association with insulin sensitivity and type 2 diabetes in Caucasians. J. Clin. Endocrinol. Metab.

[124] Osawa H., Yamada K., Onuma H., Murakami A., Ochi M., Kawata H., Nishimiya T., Niiya T., Shimizu I., Nishida W. (2004). The G/G genotype of a resistin single-nucleotide polymorphism at −420 increases type 2 diabetes mellitus susceptibility by inducing promoter activity through specific binding of Sp1/3. Am. J. Hum. Genet.

[125] Kielstein J.T., Becker B., Graf S., Brabant G., Haller H., Fliser D. (2003). Increased resistin blood levels are not associated with insulin resistance in patients with renal disease. Am. J. Kidney Dis.

[126] Patel L., Buckels A.C., Kinghorn I.J., Murdock P.R., Holbrook J.D., Plumpton C., Macphee C.H., Smith S.A. (2003). Resistin is expressed in human macrophages and directly regulated by PPARγ activators. Biochem. Biophys. Res. Commun.

[127] Qatanani M., Szwergold N.R., Greaves D.R., Ahima R.S., Lazar M.A. (2009). Macrophage-derived human resistin exacerbates adipose tissue inflammation and insulin resistance in mice. J. Clin. Investig.

[128] Mertens I., van Gaal L.F. (2002). Obesity, haemostasis and the fibrinolytic system. Obes. Rev.

[129] Juhan-Vague I., Alessi M.C., Mavri A., Morange P.E. (2003). Plasminogen activator inhibitor-1, inflammation, obesity, insulin resistance and vascular risk. J. Thromb. Haemost.

[130] Ma L.J., Mao S.L., Taylor K.L., Kanjanabuch T., Guan Y., Zhang Y., Brown N.J., Swift L.L., McGuinness O.P., Wasserman D.H. (2004). Prevention of obesity and insulin resistance in mice lacking plasminogen activator inhibitor 1. Diabetes.

[131] Liang X., Kanjanabuch T., Mao S.L., Hao C.M., Tang Y.W., Declerck P.J., Hasty A.H., Wasserman D.H., Fogo A.B., Ma L.J. (2006). Plasminogen activator inhibitor-1 modulates adipocyte differentiation. Am. J. Physiol. Endocrinol. Metab.

[132] Xu X., Wang H., Wang Z., Xiao W. (2009). Plasminogen activator inhibitor-1 promotes inflammatory process induced by cigarette smoke extraction or lipopolysaccharides in alveolar epithelial cells. Exp. Lung Res.

[133] Samal B., Sun Y., Stearns G., Xie C., Suggs S., McNiece I. (1994). Cloning and characterization of the cDNA encoding a novel human pre-B-cell colony-enhancing factor. Mol. Cell. Biol.

[134] Fukuhara A., Matsuda M., Nishizawa M., Segawa K., Tanaka M., Kishimoto K., Matsuki Y., Murakami M., Ichisaka T., Murakami H. (2005). Visfatin: A protein secreted by visceral fat that mimics the effects of insulin. Science.

[135] Revollo J.R., Körner A., Mills K.F., Satoh A., Wang T., Garten A., Dasgupta B., Sasaki Y., Wolberger C., Townsend R.R. (2007). Nampt/PBEF/Visfatin regulates insulin secretion in β cells as a systemic NAD biosynthetic enzyme. Cell Metab.

[136] Pagano C., Pilon C., Olivieri M., Mason P., Fabris R., Serra R., Milan G., Rossato M., Federspil G., Vettor R. (2006). Reduced plasma visfatin/pre B-cell colony-enhancing factor in obesity is not related to insulin resistance in humans. J. Clin. Endocrinol. Metab.

[137] Berndt J., Klöting N., Kralisch S., Kovacs P., Fasshauer M., Schön M.R., Stumvollx M., Blüher M. (2005). Plasma visfatin concentrations and fat-depot specific mRNA expression in humans. Diabetes.

[138] Fain J.N., Sacks H.S., Buehrer B., Bahouth S.W., Garrett E., Wolf R.Y., Carter R.A., Tichansky D.S., Madan A.K. (2008). Identification of omentin mRNA in human epicardial adipose tissue: Comparison to omentin in subcutaneous, internal mammary artery periadventitial and visceral abdominal depots. Int. J. Obes.

[139] Chang Y.C., Chang T.J., Lee W.J., Chuang L.M. (2010). The relationship of visfatin/pre-B-cell colony-enhancing factor/nicotinamide phosphoribosyltransferase in adipose tissue with inflammation, insulin resistance, and plasma lipids. Metabolism.

[140] Haider D.G., Schindler K., Schaller G., Prager G., Wolzt M., Ludvik B. (2006). Increased plasma visfatin concentrations in morbidly obese subjects are reduced after gastric banding. J. Clin. Endocrinol. Metab.

[141] El-Mesallamy H.O., Kassem D.H., El-Demerdash E., Amin A.I. (2011). Vaspin and visfatin/Nampt are interesting interrelated adipokines playing a role in the pathogenesis of type 2 diabetes mellitus. Metabolism.

[142] Varma V., Yao-Borengasser A., Rasouli N., Bodles A.M., Phanavanh B., Lee M.J., Starks T., Kern L.M., Spencer H.J., McGehee R.E. (2007). Human visfatin expression: Relationship to insulin sensitivity, intramyocellular lipids, and inflammation. J. Clin. Endocrinol. Metab.

[143] Kim da S., Kang S., Moon N.R., Park S. (2014). Central visfatin potentiates glucose-stimulated insulin secretion and β-cell mass without increasing serum visfatin levels in diabetic rats. Cytokine.

[144] Oki K., Yamane K., Kamei N., Nojima H., Kohno N. (2007). Circulating visfatin level is correlated with inflammation, but not with insulin resistance. Clin. Endocrinol.

[145] Quadro L., Blaner W.S., Salchow D.J., Vogel S., Piantedosi R., Gouras P., Freeman S., Cosma M.P., Colantuoni V., Gottesman M.E. (1999). Impaired retinal function and vitamin A availability in mice lacking retinol-binding protein. EMBO J.

[146] Yang Q., Graham T.E., Mody N., Preitner F., Peroni O.D., Zabolotny J.M., Kotani K., Quadro L., Kahn B.B. (2005). Serum retinol binding protein 4 contributes to insulin resistance in obesity and type 2 diabetes. Nature.

[147] Klöting N., Graham T.E., Berndt J., Kralisch S., Kovacs P., Wason C.J., Fasshauer M., Schön M.R., Stumvoll M., Blüher M. (2007). Serum retinol-binding protein is more highly expressed in visceral than in subcutaneous adipose tissue and is a marker of intra-abdominal fat mass. Cell Metab.

[148] Ost A., Danielsson A., Liden M., Eriksson U., Nystrom F.H., Stralfors P. (2007). Retinol-binding protein-4 attenuates insulin-induced phosphorylation of IRS1 and ERK1/2 in primary human adipocytes. FASEB J.

[149] Graham T.E., Yang Q., Bluher M., Hammarstedt A., Ciaraldi T.P., Henry R.R., Wason C.J., Oberbach A., Jansson P.A., Smith U. (2006). Retinol-binding protein 4 and insulin resistance in lean, obese, and diabetic subjects. N. Engl. J. Med.

[150] Gavi S., Stuart L.M., Kelly P., Melendez M.M., Mynarcik D.C., Gelato M.C., McNurlan M.A. (2007). Retinol-binding protein 4 is associated with insulin resistance and body fat distribution in nonobese subjects without type 2 diabetes. J. Clin. Endocrinol. Metab.

[151] Balagopal P., Graham T.E., Kahn B.B., Altomare A., Funanage V., George D. (2007). Reduction of elevated serum retinol binding protein in obese children by lifestyle intervention: Association with subclinical inflammation. J. Clin. Endocrinol. Metab.

[152] Nair A.K., Sugunan D., Kumar H., Anilkumar G. (2010). Case-control analysis of SNPs in GLUT4, RBP4 and STRA6: Association of SNPs in STRA6 with type 2 diabetes in a South Indian population. PLoS One.

[153] Munkhtulga L., Nakayama K., Utsumi N., Yanagisawa Y., Gotoh T., Omi T., Kumada M., Erdenebulgan B., Zolzaya K., Lkhagvasuren T. (2007). Identification of a regulatory SNP in the retinol binding protein 4 gene associated with type 2 diabetes in Mongolia. Hum. Genet.

[154] Munkhtulga L., Nagashima S., Nakayama K., Utsumi N., Yanagisawa Y., Gotoh T., Omi T., Kumada M., Zolzaya K., Lkhagvasuren T. (2010). Regulatory SNP in the RBP4 gene modified the expression in adipocytes and associated with BMI. Obesity.

[155] Yao-Borengasser A., Varma V., Bodles A.M., Rasouli N., Phanavanh B., Lee M.J., Starks T., Kern L.M., Spencer H.J., Rashidi A.A. (2007). Retinol binding protein 4 expression in humans: Relationship to insulin resistance, inflammation, and response to pioglitazone. J. Clin. Endocrinol. Metab.

[156] Ulgen F., Herder C., Kuhn M.C., Willenberg H.S., Schott M., Scherbaum W.A., Schinner S. (2010). Association of serum levels of retinol-binding protein 4 with male sex but not with insulin resistance in obese patients. Arch. Physiol. Biochem.

[157] Kos K., Wong S., Tan B., Kerrigan D., Randeva H., Pinkney J., Wilding J. (2011). Human RBP4 adipose tissue expression is gender specific and influenced by leptin. Clin. Endocrinol. (Oxf.).

[158] Tabata M., Kadomatsu T., Fukuhara S., Miyata K., Ito Y., Endo M., Urano T., Zhu H.J., Tsukano H., Tazume H. (2009). Angiopoietin-like protein 2 promotes chronic adipose tissue inflammation and obesity-related systemic insulin resistance. Cell Metab.

[159] Doi Y., Ninomiya T., Hirakawa Y., Takahashi O., Mukai N., Hata J., Iwase M., Kitazono T., Oike Y., Kiyohara Y. (2013). Angiopoietin-like protein 2 and risk of type 2 diabetes in a general Japanese population: The Hisayama study. Diabetes Care.

[160] Berg A.H., Combs T.P., Du X., Brownlee M., Scherer P.E. (2001). The adipocyte-secreted protein Acrp30 enhances hepatic insulin action. Nat. Med.

[161] Diez J.J., Iglesias P. (2003). The role of the novel adipocyte-derived hormone adiponectin in human disease. Eur. J. Endocrinol.

[162] Kern P.A., di Gregorio G.B., Lu T., Rassouli N., Ranganathan G. (2003). Adiponectin expression from human adipose tissue: Relation to obesity, insulin resistance, and tumor necrosis factor-alpha expression. Diabetes.

[163] Maeda N., Shimomura I., Kishida K., Nishizawa H., Matsuda M., Nagaretani H., Furuyama N., Kondo H., Takahashi M., Arita Y. (2002). Diet-induced insulin resistance in mice lacking adiponectin/ACRP30. Nat. Med.

[164] Kadowaki T., Yamauchi T., Kubota N., Hara K., Ueki K., Tobe K. (2006). Adiponectin and adiponectin receptors in insulin resistance, diabetes, and the metabolic syndrome. J. Clin. Investig.

[165] Ouchi N., Higuchi A., Ohashi K., Oshima Y., Gokce N., Shibata R., Akasaki Y., Shimono A., Walsh K. (2010). Sfrp5 is an anti-inflammatory adipokine that modulates metabolic dysfunction in obesity. Science.

[166] Hu Z., Deng H., Qu H. (2013). Plasma SFRP5 levels are decreased in Chinese subjects with obesity and type 2 diabetes and negatively correlated with parameters of insulin resistance. Diabetes Res. Clin. Pract.

[167] Hu W., Li L., Yang M., Luo X., Ran W., Liu D., Xiong Z., Liu H., Yang G. (2013). Circulating Sfrp5 is a signature of obesity-related metabolic disorders and is regulated by glucose and liraglutide in humans. J. Clin. Endocrinol. Metab.

[168] Tan X., Wang X., Chu H., Liu H., Yi X., Xiao Y. (2013). SFRP5 correlates with obesity and metabolic syndrome and increases after weight loss in children. Clin. Endocrinol. (Oxf.).

[169] Carstensen M., Herder C., Kempf K., Erlund I., Martin S., Koenig W., Sundvall J., Bidel S., Kuha S., Roden M. (2013). Sfrp5 correlates with insulin resistance and oxidative stress. Eur. J. Clin. Investig.

[170] Saleh J., Sniderman A.D., Cianflone K. (1999). Regulation of Plasma fatty acid metabolism. Clin. Chim. Acta.

[171] Clemente-Postigo M., Queipo-Ortuno M.I., Fernandez-Garcia D., Gomez-Huelgas R., Tinahones F.J., Cardona F. (2011). Adipose tissue gene expression of factors related to lipid processing in obesity. PLoS One.

[172] Klop B., Jukema J.W., Rabelink T.J., Castro Cabezas M. (2012). A physician’s guide for the management of hypertriglyceridemia: The etiology of hypertriglyceridemia determines treatment strategy. Panminerva Med.

[173] Klop B., Elte J.W., Cabezas M.C. (2013). Dyslipidemia in obesity: Mechanisms and potential targets. Nutrients.

[174] Guendouzi K., Jaspard B., Barbaras R., Motta C., Vieu C., Marcel Y., Chap H., Perret B., Collet X. (1999). Biochemical and physical properties of remnant-HDL2 and of pre beta 1-HDL produced by hepatic lipase. Biochemistry.

[175] Friedewald W.T., Levy R.I., Fredrickson D.S. (1972). Estimation of the concentration of low-density lipoprotein cholesterol in plasma, without use of the preparative ultracentrifuge. Clin. Chem.

[176] Contois J.H., Warnick G.R., Sniderman A.D. (2011). Reliability of low-density lipoprotein cholesterol, non-high-density lipoprotein cholesterol, and apolipoprotein B measurement. J. Clin. Lipidol.

[177] Ito Y., Fujimura M., Ohta M., Hirano T. (2011). Development of a homogeneous assay for measurement of small dense LDL cholesterol. Clin. Chem.

[178] St-Pierre A.C., Cantin B., Dagenais G.R., Mauriege P., Bernard P.-M., Despres J.P., Lamarche B. (2005). Low-density lipoprotein subfractions and the long-term risk of ischemic heart disease in men. Arterioscler. Thromb. Vasc. Biol.

[179] Rizzo M., Pernice V., Frasheri A., di Lorenzo G., Rini G.B., Spinas G.A., Berneis K. (2009). Small, dense low-density lipoproteins (LDL) are predictors of cardio- and cerebro-vascular events in subjects with the metabolic syndrome. Clin. Endocrinol. (Oxf.).

[180] Engfeldt P., Arner P. (1988). Lipolysis in human adipocytes, effects of cell size, age and of regional differences. Horm. Metab. Res.

[181] Veilleux A., Caron-Jobin M., Noël S., Laberge P.Y., Tchernof A. (2011). Visceral adipocyte hypertrophy is associated with dyslipidemia independent of body composition and fat distribution in women. Diabetes.

[182] Sam S., Haffner S., Davidson M.H., D’Agostino R.B., Feinstein S., Kondos G., Perez A., Mazzone T. (2008). Relationship of abdominal visceral and subcutaneous adipose tissue with lipoprotein particle number and size in type 2 diabetes. Diabetes.

[183] Johnson J.A., Fried S.K., Pi-Sunyer F.X., Albu J.B. (2001). Impaired insulin action in subcutaneous adipocytes from women with visceral obesity. Am. J. Physiol. Endocrinol. Metab.

[184] Despres J.P., Ferland M., Moorjani S., Nadeau A., Tremblay A., Lupien P.J., Theriault G., Bouchard C. (1989). Role of hepatic-triglyceride lipase activity in the association between intra-abdominal fat and plasma HDL cholesterol in obese women. Arteriosclerosis.

[185] Carr M.C., Hokanson J.E., Zambon A., Deeb S.S., Barrett P.H., Purnell J.Q., Brunzell J.D. (2001). The contribution of intraabdominal fat to gender differences in hepatic lipase activity and low/high density lipoprotein heterogeneity. J. Clin. Endocrinol. Metab.

[186] Cancello R., Tordjman J., Poitou C., Guilhem G., Bouillot J.L., Hugol D., Coussieu C., Basdevant A., Bar Hen A., Bedossa P. (2006). Increased infiltration of macrophages in omental adipose tissue is associated with marked hepatic lesions in morbid human obesity. Diabetes.

[187] Esteve E., Ricart W., Fernández-Real J.M. (2005). Dyslipidemia and inflammation: An evolutionary conserved mechanism. Clin. Nutr.

[188] Yang Y., Ju D., Zhang M., Yang G. (2008). Interleukin-6 stimulates lipolysis in porcine adipocytes. Endocrine.

[189] Hardardóttir I., Doerrler W., Feingold K.R., Grünfeld C. (1992). Cytokines stimulate lipolysis and decrease lipoprotein lipase activity in cultured fat cells by a prostaglandin independent mechanism. Biochem. Biophys. Res. Commun.

[190] Hardardottir I., Moser A.H., Memon R., Grunfeld C., Feingold K.R. (1994). Effects of TNF, IL-1, and the combination of both cytokines on cholesterol metabolism in Syrian hamsters. Lymphokine Cytokine Res.

[191] Kawakami M., Murase T., Ogawa H., Ishibashi S., Mori N., Takaku F., Shibata S. (1987). Human recombinant TNF suppresses lipoprotein lipase activity and stimulates lipolysis in 3T3-L1 cells. J. Biochem.

[192] Greenberg A.S., Nordan R.P., McIntosh J., Calvo J.C., Scow R.O., Jablons D. (1992). Interleukin 6 reduces lipoprotein lipase activity in adipose tissue of mice *in vivo* and in 3T3-L1 adipocytes (a possible role for interleukin 6 in cancer cachexia). Cancer Res.

[193] Rouzer C.A., Cerami A. (1980). Hypertriglyceridemia associated with Trypanosoma brucei brucei infection in rabbits: Role of defective triglyceride removal. Mol. Biochem. Parasitol.

[194] Jovinge S., Hamsten A., Tornvall P., Proudler A., Båvenholm P., Ericsson C.G., Godsland I., de Faire U., Nilsson J. (1998). Evidence for a role of tumor necrosis factor alpha in disturbances of triglyceride and glucose metabolism predisposing to coronary heart disease. Metabolism.

[195] Feingold K.R., Serio M.K., Adi S., Moser A.H., Grunfeld C. (1989). Tumor necrosis factor stimulates hepatic lipid synthesis and secretion. Endocrinology.

[196] Kawakami M., Cerami A. (1981). Studies of endotoxin-induced decrease in lipoprotein lipase activity. J. Exp. Med.

[197] Qin B., Anderson R.A., Adeli K. (2008). Tumor necrosis factor-alpha directly stimulates the overproduction of hepatic apolipoprotein B100-containing VLDL via impairment of hepatic insulin signaling. Am. J. Physiol. Gastrointest. Liver Physiol.

[198] Mohrschladt M.F., Weverling-Rijnsburger A.W., de Man F.H., Stoeken D.J., Sturk A., Smelt A.H., Westendorp R.G. (2000). Hyperlipoproteinemia affects cytokine production in whole blood samples *ex vivo*. The influence of lipid-lowering therapy. Atherosclerosis.

[199] Jonkers I.J., Mohrschladt M.F., Westendorp R.G., van der Laarse A., Smelt A.H. (2002). Severe hypertriglyceridemia with insulin resistance is associated with systemic inflammation (reversal with bezafibrate therapy in a randomized controlled trial). Am. J. Med.

[200] Nappo F., Esposito K., Cioffi M., Giugliano G., Molinari A.M., Paolisso G., Marfella R., Giugliano D. (2002). Postprandial endothelial activation in healthy subjects and in type 2 diabetic patients (role of fat and carbohydrate meals). J. Am. Coll. Cardiol.

[201] Van Exel E., Gussekloo J., de Craen A.J., Frolich M., Bootsma-van der Wiel A., Westendorp R.G., Leiden 85 Plus Study (2002). Low production capacity of interleukin-10 associates with the metabolic syndrome and type 2 diabetes: The Leiden 85-Plus Study. Diabetes.

[202] Grunfeld C., Dinarello C.A., Feingold K.R. (1991). Tumor necrosis factor-alpha, interleukin-1, and interferon alpha stimulate triglyceride synthesis in HepG2 cells. Metabolism.

[203] Fernandez-Real J.M., Gutierrez C., Ricart W., Castineira M.J., Vendrell J., Richart C. (1999). Plasma levels of the soluble fraction of tumor necrosis factor receptors 1 and 2 are independent determinants of plasma cholesterol and LDL-cholesterol concentrations in healthy subjects. Atherosclerosis.

[204] Skoog T., Dichtl W., Boquist S., Skoglund-Andersson C., Karpe F., Tang R., Bond M.G., de Faire U., Nilsson J., Eriksson P. (2002). Plasma tumour necrosis factor-alpha and early carotid atherosclerosis in healthy middle-aged men. Eur. Heart J.

[205] Mizia-Stec K., Zahorska-Markiewicz B., Mandecki T., Janowska J., Szulc A., Jastrzekbska-Maj E., Gasior Z. (2003). Hyperlipidaemias and serum cytokines in patients with coronary artery disease. Acta Cardiol.

[206] Saez Y., Vacas M., Santos M., Saez de Lafuente J.P., Sagastagoitia J.D., Molinero E., Iriarte J.A. (2012). Relation of high-density lipoprotein cholesterol and apoprotein A1 levels with presence and severity of coronary obstruction. ISRN Vasc. Med.

[207] Ettinger W.H., Miller L.A., Smith T.K., Parks J.S. (1992). Effect of interleukin-1 alpha on lipoprotein lipids in cynomolgus monkeys (comparison to tumor necrosis factor). Biochim. Biophys. Acta.

[208] Ettinger W.H., Miller L.D., Albers J.J., Smith T.K., Parks J.S. (1990). Lipopolysaccharide and tumor necrosis factor cause a fall in plasma concentration of lecithin (cholesterol acyltransferase in cynomolgus monkeys). J. Lipid Res.

[209] Memon R.A., Grunfeld C., Moser A.H., Feingold K.R. (1993). Tumor necrosis factor mediates the effects of endotoxin on cholesterol and triglyceride metabolism in mice. Endocrinology.

[210] Feingold K.R., Pollock A.S., Moser A.H., Shigenaga J.K., Grunfeld C. (1995). Discordant regulation of proteins of cholesterol metabolism during the acute phase response. J. Lipid Res.

[211] Ruan X.Z., Varghese Z., Fernando R., Moorhead J.F. (1998). Cytokine regulation of low-density lipoprotein receptor gene transcription in human mesangial cells. Nephrol. Dial. Transplant.

[212] Ruan X.Z., Varghese Z., Powis S.H., Moorhead J.F. (2001). Dysregulation of LDL receptor under the influence of inflammatory cytokines (a new pathway for foam cell formation). Kidney Int.

[213] Ruan X.Z., Moorhead J.F., Fernando R., Wheeler D.C., Powis S.H., Varghese Z. (2004). Regulation of lipoprotein trafficking in the kidney (role of inflammatory mediators and transcription factors). Biochem. Soc. Trans.

[214] Bartolome N., Rodriguez L., Martinez M.J., Ochoa B., Chico Y. (2007). Upregulation of apolipoprotein B secretion, but not lipid, by tumor necrosis factor-alpha in rat hepatocyte cultures in the absence of extracellular fatty acids. Ann. N. Y. Acad. Sci.

[215] Homma Y. (2004). Predictors of atherosclerosis. J. Atheroscler. Thromb.

[216] Crowl R.M., Stoller T.J., Conroy R.R., Stoner C.R. (1991). Induction of phospholipase A2 gene expression in human hepatoma cells by mediators of the acute phase response. J. Biol. Chem.

[217] Kugiyama K., Ota Y., Takazoe K., Moriyama Y., Kawano H., Miyao Y., Sakamoto T., Soejima H., Ogawa H., Doi H. (1999). Circulating levels of secretory type II phospholipase A(2) predict coronary events in patients with coronary artery disease. Circulation.

[218] O’Brien K.D., Chait A. (2006). Serum amyloid A: The “other” inflammatory protein. Curr. Atheroscler. Rep.

[219] Poitou C., Viguerie N., Cancello R., de Matteis R., Cinti S., Stich V., Coussieu C., Gauthier E., Courtine M., Zucker J.D. (2005). Serum amyloid A: Production by human white adipocyte and regulation by obesity and nutrition. Diabetologia.

[220] Fasshauer M., Klein J., Kralisch S., Klier M., Lossner U., Bluher M., Paschke R. (2004). Serum amyloid A3 expression is stimulated by dexamethasone and interleukin-6 in 3T3–L1 adipocytes. J. Endocrinol.

[221] Yang R.Z., Lee M.J., Hu H., Pollin T.I., Ryan A.S., Nicklas B.J., Snitker S., Horenstein R.B., Hull K., Goldberg N.H. (2006). Acute-phase serum amyloid A: An inflammatory adipokine and potential link between obesity and its metabolic complications. PLoS Med.

[222] Chen C.H., Wang P.H., Liu B.H., Hsu H.H., Mersmann H.J., Ding S.T. (2008). Serum amyloid A protein regulates the expression of porcine genes related to lipid metabolism. J. Nutr.

[223] Cai L., de Beer M.C., de Beer F.C., van der Westhuyzen D.R. (2005). Serum amyloid A is a ligand for scavenger receptor class B type I and inhibits high density lipoprotein binding and selective lipid uptake. J. Biol. Chem.

[224] Rigotti A., Miettinen H.E., Krieger M. (2003). The role of the high-density lipoprotein receptor SR-BI in the lipid metabolism of endocrine and other tissues. Endocr. Rev.

[225] Lewis K.E., Kirk E.A., McDonald T.O., Wang S., Wight T.N., O’Brien K.D., Chait A. (2004). Increase in serum amyloid a evoked by dietary cholesterol is associated with increased atherosclerosis in mice. Circulation.

[226] Ouchi N., Kihara S., Arita Y., Maeda K., Kuriyama H., Okamoto Y., Hotta K., Nishida M., Takahashi M., Nakamura T. (1999). Novel modulator for endothelial adhesion molecules: Adipocyte-derived plasma protein adiponectin. Circulation.

[227] Matsubara M., Maruoka S., Katayose S. (2002). Decreased plasma adiponectin concentrations in women with dyslipidemia. J. Clin. Endocrinol. Metab.

[228] Okamoto Y., Kihara S., Funahashi T., Matsuzawa Y., Libby P. (2006). Adiponectin: A key adipocytokine in metabolic syndrome. Clin. Sci. (Lond.).

[229] Yamauchi T., Kamon J., Minokoshi Y., Ito Y., Waki H., Uchida S., Yamashita S., Noda M., Kita S., Ueki K. (2002). Adiponectin stimulates glucose utilization and fatty-acid oxidation by activating AMP-activated protein kinase. Nat. Med.

[230] Berneis K.K., Krauss R.M. (2002). Metabolic origins and clinical significance of LDL heterogeneity. J. Lipid Res.

[231] Schneider J.G., von Eynatten M., Schiekofer S., Nawroth P.P., Dugi K.A. (2005). Low plasma adiponectin levels are associated with increased hepatic lipase activity*in vivo*. Diabetes Care.

[232] Matsuura F., Oku H., Koseki M., Sandoval J.C., Yuasa-Kawase M., Tsubakio-Yamamoto K., Masuda D., Maeda N., Tsujii K., Ishigami M. (2007). Adiponectin accelerates reverse cholesterol transport by increasing high density lipoprotein assembly in the liver. Biochem. Biophys. Res. Commun.

[233] Oku H., Matsuura F., Koseki M., Sandoval J.C., Yuasa-Kawase M., Tsubakio-Yamamoto K., Masuda D., Maeda N., Ohama T., Ishigami M. (2007). Adiponectin deficiency suppresses ABCA1 expression and ApoA-I synthesis in the liver. FEBS Lett.

[234] Chang C.Y., Chen M.J., Yang W.S., Yeh C.Y., Ho H.N., Chen S.U., Yang Y.S. (2012). Hypoadiponectinemia: A useful marker of dyslipidemia in women with polycystic ovary syndrome. Taiwan J. Obstet. Gynecol.

[235] Angulo P. (2002). Nonalcoholic fatty liver disease. N. Engl. J. Med.

[236] Starley B.Q., Calcagno C.J., Harrison S.A. (2010). Nonalcoholic fatty liver disease and hepatocellular carcinoma: A weighty connection. Hepatology.

[237] Lazo M., Clark J.M. (2008). The epidemiology of nonalcoholic fatty liver disease: A global perspective. Semin Liver Dis.

[238] Tarantino G., Savastano S., Colao A. (2010). Hepatic steatosis, low-grade chronic inflammation and hormone/growth factor/adipokine imbalance. World J. Gastroenterol.

[239] Day C.P., James O.F. (1998). Steatohepatitis: A tale of two “hits”?. Gastroenterology.

[240] Tilg H., Moschen A.R. (2010). Evolution of inflammation in nonalcoholic fatty liver disease: The multiple parallel hits hypothesis. Hepatology.

[241] Polyzos S.A., Kountouras J., Zavos C., Deretzi G. (2012). Nonalcoholic fatty liver disease: Multimodal treatment options for a pathogenetically multiple-hit disease. J. Clin. Gastroenterol.

[242] Bradbury M.W., Berk P.D. (2004). Lipid metabolism in hepatic steatosis. Clin. Liver Dis.

[243] Koteish A., Diehl A.M. (2001). Animal models of steatosis. Semin. Liver Dis.

[244] Lambert J.E., Ramos-Roman M.A., Browning J.D., Parks E.J. (2014). Increased de novo lipogenesis is a distinct characteristic of individuals with nonalcoholic Fatty liver disease. Gastroenterology.

[245] Nielsen S., Guo Z., Johnson C.M., Hensrud D.D., Jensen M.D. (2004). Splanchnic lipolysis in human obesity. J. Clin. Investig.

[246] Heilbronn L., Smith S.R., Ravussin E. (2004). Failure of fat cell proliferation, mitochondrial function and fat oxidation results in ectopic fat storage, insulin resistance and type II diabetes mellitus. Int. J. Obes. Relat. Metab. Disord.

[247] Roden M. (2006). Mechanisms of disease: Hepatic steatosis in type 2 diabetes—Pathogenesis and clinical relevance. Nat. Clin. Pract. Endocrinol. Metab.

[248] Montague C.T., O’Rahilly S. (2000). The perils of portliness: Causes and consequences of visceral adiposity. Diabetes.

[249] Donnelly K.L., Smith C.I., Schwarzenberg S.J., Jessurun J., Boldt M.D., Parks E.J. (2005). Sources of fatty acids stored in liver and secreted via lipoproteins in patients with nonalcoholic fatty liver disease. J. Clin. Investig.

[250] Parks E.J., Hellerstein M.K. (2006). Thematic review series: Patient-oriented research. Recent advances in liver triacylglycerol and fatty acid metabolism using stable isotope labeling techniques. J. Lipid Res.

[251] Harrison S.A., Day C.P. (2007). Benefits of lifestyle modification in NAFLD. Gut.

[252] Shklyaev S., Aslanidi G., Tennant M., Prima V., Kohlbrenner E., Kroutov V., Campbell-Thompson M., Crawford J., Shek E.W., Scarpace P.J. (2003). Sustained peripheral expression of transgene adiponectin offsets the development of diet-induced obesity in rats. Proc. Natl. Acad. Sci. USA.

[253] Xu A., Wang Y., Keshaw H., Xu L.Y., Lam K.S., Cooper G.J. (2003). The fat-derived hormone adiponectin alleviates alcoholic and nonalcoholic fatty liver diseases in mice. J. Clin. Investig.

[254] Schindhelm R.K., Diamant M., Dekker J.M., Tushuizen M.E., Teerlink T., Heine R.J. (2006). Alanine aminotransferase as a marker of non-alcoholic fatty liver disease in relation to type 2 diabetes mellitus and cardiovascular disease. Diabetes Metab. Res. Rev.

[255] Masaki T., Chiba S., Tatsukawa H., Yasuda T., Noguchi H., Seike M., Yoshimatsu H. (2004). Adiponectin protects LPS-induced liver injury through modulation of TNF-alpha in KK-Ay obese mice. Hepatology.

[256] Kamada Y., Tamura S., Kiso S., Matsumoto H., Saji Y., Yoshida Y., Fukui K., Maeda N., Nishizawa H., Nagaretani H. (2003). Enhanced carbon tetrachloride-induced liver fibrosis in mice lacking adiponectin. Gastroenterology.

[257] Hui J.M., Hodge A., Farrell G.C., Kench J.G., Kriketos A., George J. (2004). Beyond insulin resistance in NASH: TNF-alpha or adiponectin?. Hepatology.

[258] López-Bermejo A., Botas P., Funahashi T., Delgado E., Kihara S., Ricart W., Fernández-Real J.M. (2004). Adiponectin, hepatocellular dysfunction and insulin sensitivity. Clin. Endocrinol. (Oxf.).

[259] Targher G., Bertolini L., Scala L., Poli F., Zenari L., Falezza G. (2004). Decreased plasma adiponectin concentrations are closely associated with nonalcoholic hepatic steatosis in obese individuals. Clin. Endocrinol. (Oxf.).

[260] Kaser S., Moschen A., Cayon A., Kaser A., Crespo J., Pons-Romero F., Ebenbichler C.F., Patsch J.R., Tilg H. (2005). Adiponectin and its receptors in non-alcoholic steatohepatitis. Gut.

[261] Stefan N., Machicao F., Staiger H., Machann J., Schick F., Tschritter O., Spieth C., Weigert C., Fritsche A., Stumvoll M. (2005). Polymorphisms in the gene encoding adiponectin receptor 1 are associated with insulin resistance and high liver fat. Diabetologia.

[262] Minokoshi Y., Kim Y.B., Peroni O.D., Fryer L.G., Muller C., Carling D., Kahn B.B. (2002). Leptin stimulates fatty-acid oxidation by activating AMP-activated protein kinase. Nature.

[263] Kakuma T., Lee Y., Higa M., Wang Z.W., Pan W., Shimomura I., Unger R.H. (2000). Leptin, troglitazone, and the expression of sterol regulatory element binding proteins in liver and pancreatic islets. Proc. Natl. Acad. Sci. USA.

[264] Lee Y., Yu X., Gonzales F., Mangelsdorf D.J., Wang M.Y., Richardson C., Witters L.A., Unger R.H. (2002). PPAR alpha is necessary for the lipogenic action of hyperleptinemia on white adipose and liver tissue. Proc. Natl. Acad. Sci. USA.

[265] Serin E., Ozer B., Gümürdülü Y., Kayaselçuk F., Kul K., Boyacioğlu S. (2003). Serum leptin level can be a negative marker of hepatocyte damage in nonalcoholic fatty liver. J. Gastroenterol.

[266] Poordad F.F. (2004). The role of leptin in NAFLD contender or pretender?. J. Clin. Gastroenterol.

[267] Tobe K., Ogura T., Tsukamoto C., Imai A., Matsuura K., Iwasaki Y., Shimomura H., Higashi T., Tsuji T. (1999). Relationship between serum leptin and fatty liver in Japanese male adolescent university students. Am. J. Gastroenterol.

[268] Marra F. (2002). Leptin and liver fibrosis: A matter of fat. Gastroenterology.

[269] Cao Q., Mak K.M., Ren C., Lieber C.S. (2004). Leptin stimulates tissue inhibitor of metalloproteinase-1 in human hepatic stellate cells: Respective roles of the JAK/STAT and JAK-mediated H2O2-dependent MAPK pathways. J. Biol. Chem.

[270] Saxena N.K., Titus M.A., Ding X., Floyd J., Srinivasan S., Sitaraman S.V., Anania F.A. (2004). Leptin as a novel profibrogenic cytokine in hepatic stellate cells: Mitogenesis and inhibition of apoptosis mediated by extracellular regulated kinase (Erk) and Akt phosphorylation. FASEB J.

[271] Banerjee R.R., Lazar M.A. (2003). Resistin: Molecular history and prognosis. J. Mol. Med.

[272] Pagano C., Soardo G., Pilon C., Milocco C., Basan L., Milan G., Donnini D., Faggian D., Mussap M., Plebani M. (2006). Increased serum resistin in nonalcoholic fatty liver disease is related to liver disease severity and not to insulin resistance. J. Clin. Endocrinol. Metab.

[273] Crespo J., Cayon A., Fernandez-Gil P., Hernández-Guerra M., Mayorga M., Domínguez-Díez A., Fernández-Escalante J.C., Pons-Romero F. (2001). Gene expression of tumor necrosis factor alpha and TNF-receptors, p55 and p75, in nonalcoholic steatohepatitis patients. Hepatology.

[274] Ding W.X., Yin X.M. (2004). Dissection of the multiple mechanisms of TNF-alpha-induced apoptosis in liver injury. J. Cell. Mol. Med.

[275] Manco M., Marcellini M., Giannone G., Nobili V. (2007). Correlation of serum TNF-alpha levels and histologic liver injury scores in pediatric nonalcoholic fatty liver disease. Am. J. Clin. Pathol.

[276] Sanal M.G. (2008). The blind men see the elephant-the many faces of fatty liver disease. World J. Gastroenterol.

[277] Saleh J., Christou N., Cianflone K. (1999). Regional specificity of ASP binding in human adipose tissue. Am. J. Physiol.

[278] Massiera F., Bloch-Faure M., Ceiler D., Murakami K., Fukamizu A., Gasc J.M., Quignard-Boulange A., Negrel R., Ailhaud G., Seydoux J. (2001). Adipose angiotensinogen is involved in adipose tissue growth and blood pressure regulation. FASEB J.

[279] Umemura S., Nyui N., Tamura K., Hibi K., Yamaguchi S., Nakamaru M., Ishigami T., Yabana M., Kihara M., Inoue S. (1997). Plasma angiotensinogen concentrations in obese patients. Am. J. Hypertens.

[280] Dixon J.B., Bhathal P.S., Jonsson J.R., Dixon A.F., Powell E.E., O’Brien P.E. (2003). Pro-fibrotic polymorphisms predictive of advanced liver fibrosis in the severely obese. J. Hepatol.

